# Transient DNMT1 suppression reveals hidden heritable marks in the genome

**DOI:** 10.1093/nar/gku1386

**Published:** 2015-01-10

**Authors:** Serge McGraw, Jacques X. Zhang, Mena Farag, Donovan Chan, Maxime Caron, Carolin Konermann, Christopher C. Oakes, K. Naga Mohan, Christoph Plass, Tomi Pastinen, Guillaume Bourque, J. Richard Chaillet, Jacquetta M. Trasler

**Affiliations:** 1 Departments of Pediatrics, Human Genetics and Pharmacology & Therapeutics, McGill University and the Research Institute of the McGill University Health Centre at the Montreal Children's Hospital, Montreal, QC H3Z 2Z3, Canada; 2 Department of Human Genetics, McGill University and Genome Quebec Innovation Centre, Montreal, QC H3A 1A4, Canada; 3 Division of Epigenomics and Cancer Risk Factors, German Cancer Research Center, Heidelberg 69120, Germany; 4 Department of Biological Sciences, Birla Institute of Technology and Science Pilani, Hyderabad 500 078, India; 5 Department of Microbiology and Molecular Genetics, University of Pittsburgh, Pittsburgh, PA 15213-3005, USA

## Abstract

Genome-wide demethylation and remethylation of DNA during early embryogenesis is essential for development. Imprinted germline differentially methylated domains (gDMDs) established by sex-specific methylation in either male or female germ cells, must escape these dynamic changes and sustain precise inheritance of both methylated and unmethylated parental alleles. To identify other, gDMD-like sequences with the same epigenetic inheritance properties, we used a modified embryonic stem (ES) cell line that emulates the early embryonic demethylation and remethylation waves. Transient DNMT1 suppression revealed gDMD-like sequences requiring continuous DNMT1 activity to sustain a highly methylated state. Remethylation of these sequences was also compromised *in vivo* in a mouse model of transient DNMT1 loss in the preimplantation embryo. These novel regions, possessing heritable epigenetic features similar to imprinted-gDMDs are required for normal physiological and developmental processes and when disrupted are associated with disorders such as cancer and autism spectrum disorders. This study presents new perspectives on DNA methylation heritability during early embryo development that extend beyond conventional imprinted-gDMDs.

## INTRODUCTION

It is now well recognized that there are many epigenetic and functional states of the eukaryotic nuclear genome—any given region, within or outside genes, can take on different epigenetic states and these are related to different gene functions. The most well characterized epigenetic modification in the mammalian genome is the methylation of cytosine residues in the cytosine-guanine (CG) sequence context. Methylation is often associated with repression of gene activity, whereas the lack of methylation is associated with gene expression. The majority (∼70–80%) of CG dinucleotides are methylated in adult somatic cells, however, the developmental and molecular origins of this methylation are only partly understood. Most of this DNA methylation is established *de novo* in peri-implantation embryos through the action of the DNMT3a and DNMT3b cytosine methyltransferases (1,2). An interesting minority of sequences, imprinted genes, acquire methylation earlier, in a parent-of-origin-dependent manner on germline differentially methylated domains (gDMDs) (3–5). For each gDMD, one parental allele becomes methylated during gametogenesis by the action of the DNMT3 family, whereas the opposite parental allele remains unmethylated. The methylated and unmethylated gDMD allelic states are then inherited following fertilization. The importance of epigenetic inheritance, the strict inheritance of both the methylated and unmethylated state, for imprinted gDMDs is best shown by *Dnmt1* gene targeting studies (6–9).

DNMT1 activity is critically important for sustaining a high level of genomic methylation and the inheritance (maintenance) of gDMD methylation. However, DNMT1's mechanism in maintaining a high level of genomic methylation on non-imprinted sequences may be different from its action on imprinted genes. This distinction is evident in many studies in which re-expression of DNMT1 in embryonic stem (ES) cells lacking DNMT1 for an extended period led to non-imprinted sequence methylation but not imprinted gDMD methylation (10–12). These ES cell findings are mirrored *in vivo* in the *Dnmt1o* knockout mouse model where loss of gDMD methylation at a single stage of preimplantation is not recovered postimplantation (7,13–16). Both the recovery of non-imprinted genomic methylation in these experimental systems and maintenance of genomic methylation in many cell types probably require cooperativity between a DNMT3 *de novo* methyltransferase and DNMT1 (17–19).

The immunity of unmethylated DMD alleles to *de novo* methylation outside of the specific parental germ line initially setting their methylated state is a critically important defining gDMD attribute—without this immunity both gDMD alleles would become equally methylated, negating the gDMD and its vital control of gene expression and development. The other important attribute is the direct inheritance of the methylated parental allele by DNMT1. In addition to DNMT1, *trans*-acting factors such as the KRAB (Krüppel-associated box-containing) zinc finger protein system including ZFP57 and KAP1/TRIM28, are also critical in maintaining gDMD methylation specifically during preimplnatation development (20–24). The dual nature of gDMDs, specifically the co-inheritance of methylated and unmethylated alleles, provides a powerful epigenetic device for mammalian development. Beyond the extremely potent dose-regulating mechanism, imprinted genes affect cell survival and differentiation and are associated with cell-signaling pathways. The importance of appropriate expression of these genes is highlighted by the developmental abnormalities resulting from general or specific loss of imprinting.

The ability of identical sequences to take on two distinct and heritable epigenetic states may be confined to gDMDs and gDMD-like sequences. Allele-specific DNA methylation (ASM) can also be found in and around non-imprinted genes in both mouse and human (25,26), but contrary to imprinted genes, differences in DNA sequence and nucleosomal composition between the two alleles appear to control this allelic asymmetry. If, across the genome, epigenetic structures are determined by DNA sequence, then most epigenetic states are not inherited—they only appear to be so because the epigenetic states are driven by DNA sequence, and DNA sequence is accurately inherited by the high-fidelity process of DNA replication. Therefore, identifying actual gDMD-like sequences is especially important because of their epigenetically heritable nature and possible vital developmental functions. Moreover, gDMD-like sequences may acquire their initial methylation after fertilization in early embryos and may not be discernible by germline allele-specific methylation.

**Figure 1.**
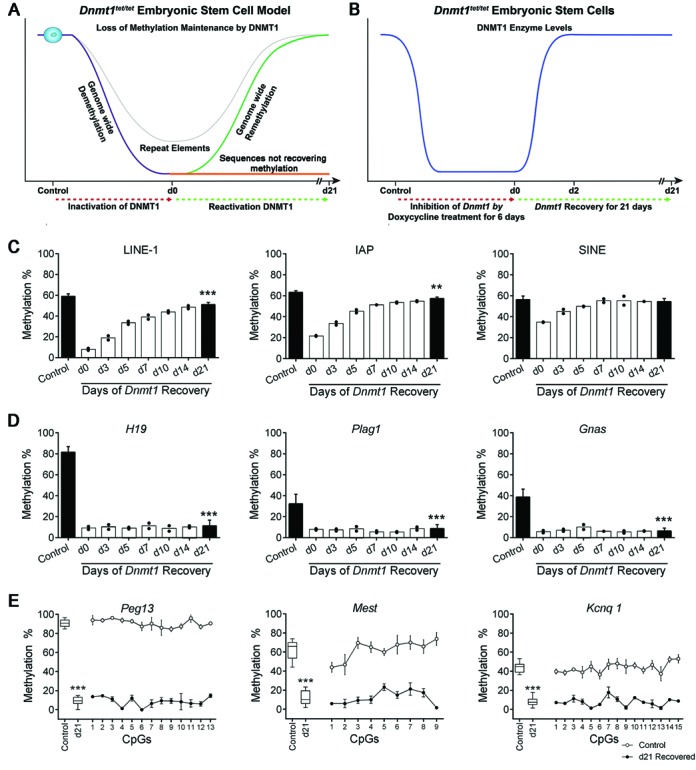
Modeling the major DNA methylation transition in early embryos using embryonic stem cells. (**A**) Inactivation of DNMT1 in *Dnmt1^tet/tet^* ES cells generates a genome wide phase of passive demethylation (purple line) by preventing the methylation maintenance process. Using this ES cell model, we predict that heritable imprinted marks (gDMDs) will lose their methylation following suppression of DNMT1 and not recover their original levels upon reactivation of DNMT1. We also predict that regions of the genome will maintain variable levels of DNA methylation in the absence of DNMT1. We expect that soon after DNMT1 re-expression, methylation levels in most regions of the genome will recover their original levels (green and gray line), exposing regions that do not recover their methylation (orange line). (**B**) In the course of inhibition of *Dnmt1* expression by doxycycline in *Dnmt1^tet/tet^* ES cells (red dotted line), DNMT1 (blue line) protein was undetectable after 3 days (10). Subsequent to *Dnmt1* reactivation (green dotted line), DNMT1 protein levels returned to initial levels after 2 days (blue line). (C–E) DNA methylation of repeat sequences and imprinted genes following the depletion and re-expression of *Dnmt1* in *Dnmt1^tet/tet^* ES cells after 21 days. Methylation levels are shown as average methylation ± SD (control (*n* = 5) versus d21 (*n* = 6)). Methylation levels were also quantified from d0 to d14 (*n* = 2) in order to visualize how methylation patterns were restored following the reactivation of *Dnmt1* (C and D). (**C**) Repeat sequences (LINE-1, IAP, SINE) were able to regain most or all of their original methylation levels following the reactivation of *Dnmt1*. (**D**) Germline-DMDs (gDMDs) of imprinted genes (*H19, Plag1, Gnas*) did not recover methylation following the reactivation of *Dnmt1*. (**E**) Levels of methylation at individual CpG sites in specific imprinted gene (*Peg13, Mest, Kcnq1*) gDMDs showed similar levels across a number of CpGs. (C–E) Quantified by Sequenom MassArray. Control versus d21 *Dnmt1^tet/tet^* ES cells: ^***^P < 0.005, ^**^P < 0.01. See also Supplementary Figures S1 and S2.

Here, we set out to model the effects of loss of maintenance methylation activity during preimplantation development on heritable DNA methylation. From previous work, loss of DNMT1o at just the eight-cell stage results in partial loss of gDMD methylation. We postulate that a complete preimplantation maintenance methylation loss would lead to a complete loss of gDMD methylation and possibly loss of methylation on other non-gDMD sequences that carry important heritable epigenetic information. To test this, we investigated changes in methylation following a controlled transient yet complete loss of DNMT1 activity in *Dnmt1^tet/tet^* ES cells. We used a modified ES cell line that emulates the demethylation and remethylation phases occurring in early embryogenesis to identify two types of endogenous genomic DNA sequences, those that recover their methylation following re-expression of DNMT1 after a transient loss in expression, and those that do not recover their methylation (Figure 1A and B). The former type presumably regain methylation due to combined *de novo* and DNMT1 activities, and whose methylation in ES cells does not require a bona fide epigenetic inheritance mechanism. The latter type does not regain methylation because their methylation in ES cells is due solely to an epigenetic inheritance process. Some of our previous findings suggest that a large disordered region in the amino terminus of DNMT1 is involved in determining these two types of DNMT1 activities (10). In recent years, technological innovation has permitted the description of DNA methylome maps that provide accurate representations of DNA methylation establishment in female and male germlines (27–29). These catalogues furthered our understanding on heritable marks laid down on the maternal and paternal germ cell genomes. However, our knowledge of the molecular mechanisms mediating the perpetuation of non-imprinted methylation remains unclear. Because of this, we set out to determine if there are gDMD-like sequences with the cardinal attributes of gDMDs. Our findings may be relevant to the issue of possible transgenerational epigenetic inheritance.

## MATERIALS AND METHODS

### Dnmt1^tet/tet^ ES cells

The *Dnmt1^tet/tet^* ES cell model was previously described in (10). Briefly, the two endogenous *Dnmt1* alleles of a male R1 ES cell line were sequentially targeted in order to introduce a tetracycline-controllable expression system (*Tet-off*). Classical homologous recombination incorporated the constructs 30 nucleotides upstream of the initiation codon of Dnmt1s. Addition of 2 ug/ml doxycycline (DOX), a tetracycline derivative, to the culture medium for 3 days results in a total lack of DNMT1 protein in *Dnmt1^tet/tet^* ES cells (10). The treatment was applied for a total of 6 days and no *Dnmt1* mRNA was detectable (Supplementary Figure S1). Within 48 h of the removal of DOX from the culture medium, the DNMT1 protein regains its expression. Samples of *Dnmt1^tet/tet^* ES cells were collected prior to (control) and immediately following DOX treatment (d0), as well as at various time points over a 21-day period of recovery from DOX exposure (d3–d21).

### Dnmt1^Δ1o/Δ1o^ conceptuses

The mutant *Dnmt1^Δ1o^* allele was generated by targeted deletion of exon 1o of the mouse *Dnmt1* gene (7). Homozygous *Dnmt1^Δ1o/Δ1o^* females or wild-type *Dnmt1^+/+^* females were crossed to wild-type 129/Sv males (Charles River Canada, St-Constant, QC, Canada). The presence of a vaginal plug was designated 0.5 days post-coitum (dpc). Embryos and placentae were collected from the uteri at 9.5dpc. All animal procedures were carried out in accordance with the Canadian Council of Animal Care.

### Genomic DNA extraction

Genomic DNA was isolated from *Dnmt1^tet/tet^* ES cells and 9.5dpc wild-type and *Dnmt1o*-deficient mouse embryos and placentae using the QIAamp DNA mini Kit (Qiagen, Mississauga, ON, Canada). The gender of samples was assessed by PCR using the male specific *Zfy1* primers and *Myog* as an internal control. High-molecular weight DNA for RLGS was isolated using proteinase K digestion and phenol-chloroform extraction.

### DNA methylation analyses

Quantitative measurement of the DNA methylation levels on CpG dinucleotides was accomplished on isolated genomic DNA that was subjected to bisulfite treatment using the EpiTect Bisulfite kit (Qiagen #59104). The various candidate regions, imprinted gDMDs or repeat elements were amplified using primers specific to quantitative mass spectrometric analysis (*Sequenom's EpiTYPER* technology, San Diego, CA, USA) or pyrosequencing applications. For the Sequenom assay, amplified DNA was transcribed *in vitro*, cleaved using RNAse A and subjected to MALDI-TOF mass spectrometry analysis. DNA methylation standards (0–100%) were used to control for PCR amplification bias. Equation fitting algorithms based on the R statistic were used for data correction and potential PCR bias (30). Pyrosequencing was performed as previously described (31). Amplified sequences were sequenced using the PyroMark Q24 kit (Qiagen #970802) and the PyroMarkR Q24 Vacuum Workstation (Qiagen, Valencia, CA, USA) using the manufacturer's protocol. Primers are listed in Supplementary Table S5.

Restriction landmark genomic scanning (RLGS) was used to assess genome-wide methylation in *Dnmt1^tet/tet^* ES cells collected at various time points previous to (control; no-DOX) or following the recovery of 6 days of DOX exposure (d0, d3, d5, d7, d10, d14, d21). RLGS was performed on the extracted DNA as described (16,32,33). RLGS gels were dried and exposed to autoradiographic film (GE Healthcare/Amersham) and phosphor imaging. Each spot was analyzed and intensities were compared. Methylation scores ranged from 0% methylation (intensely dark spot) to 100% methylation (absence of spot). Spot identities were determined by using the virtual RLGS method (34).

The methyl-CpG immunoprecipitation (MCIp) technique was performed as previously described (35–38). Using 60 μg of a recombinant methyl-CpG binding domain-based protein 2 (MBD2), highly CpG methylated DNA was enriched from 3 μg genomic DNA from control (no-Dox) (*n* = 2) and d21 recovered (*n* = 2) *Dnmt1^tet/tet^* ES cells. The enriched DNA was directly applied to fluorescent labeling and oligonucleotide microarray hybridization (Mouse CpG Island Arrays, Agilent) (2×: control versus d21) without an additional amplification step. Data analysis and identification of probes most frequently detected as positive within a CGI were performed as previously described (36), with the top 2% of probes with respect to positive or negative *M*-values and common in duplicate experiments, considered hypermethylated or hypomethylated probes.

Reduced representation bisulfite sequencing (RRBS) libraries were generated according to previously published protocols using the gel-free technique (39,40). Briefly, 500 ng of DNA from each sample (*Dnmt1^tet/tet^* ES cells; control no-Dox, d0 and d21) was used in the RRBS experiments and the 10 samples were multiplexed. Samples were then used in paired end sequencing in one lane of a HiSeq 2000 sequencer (Illumina). Initial data processing, methylation calls and alignment of reads used the software pipeline bsmap version 2.6 (41). For analysis and statistics of regions that were differentially methylated the software MethylKit (version 0.5.3) was used (42); this software uses the Benjamini–Hochberg FDR procedure (*P*-value threshold of *q* = 0.01). For the analysis, specific parameters were chosen including 100 bp step-wise tiling windows, 1 CpG minimum per tile and a minimum 20× CpG coverage of each tile per sample. The methylation level of a 100-bp tile was the average of all single CpGs within the tile, and the methylation level reported for a sample was the average methylation level across replicates. The average number of CpGs/100 bp tile was 3.5 for non-CGI regions (total = 9654) and 7 for CGI regions (total = 230) for the control versus d21 comparison. Significant DNA methylation changes were designated as ±≥20% average differences between groups of replicates. Differentially methylated sequences were annotated using HOMER version 3.51 (43). Correlation values between samples on a per-tile basis were determined using the pair-wise Pearson's correlation calculation. DNA binding motifs were analyzed using HOMER on 100 bp tiles ± 100 bp.

## DATA ACCESS

The sequencing data from this study have been submitted to the European Nucleotide Archive (ENA) under project accession number PRJEB6698.

## RESULTS

### Repeat sequences recover DNA methylation patterns following transient loss of DNMT1

Throughout the embryonic reprogramming wave, repetitive elements (REs) demonstrate variable but significant degrees of retention of DNA methylation patterns (29,44,45). Using quantitative Sequenom MassArray, the methylation status of various REs (LINE-1, IAP, SINE, Minor Satellite and Gamma Satellite) was determined before and following the pharmacological inactivation of *Dnmt1* in our ES cell reprogramming model (Figure 1A and B). Upon suppression of DNMT1 (day 0—d0), we detected a substantial reduction in DNA methylation levels for all types of REs (Figure 1C, Supplementary Figure S2). However, except for LINE-1, REs retained considerable CpG methylation (∼22–35%) at d0. Following re-expression of DNMT1, methylation levels increased until they reached their original levels by d21, except for LINE-1 and IAP where slight but significant reductions were observed (8% *P* < 0.005 and 6% *P* <0.01 respectively). Thus, our ES cell methylation reprogramming model shows similar patterns of methylation retention and retrieval on REs as those observed during the *in vivo* embryonic reprogramming wave.

### Imprinted gene gDMD methylation is lost in ES cells transiently lacking DNMT1

During the early embryonic reprogramming wave, gDMDs are the only sequences known to require complete methylation maintenance and embryonic loss of DNMT1 function leads to permanent loss of methylation patterns on the gDMDs of imprinted genes (8,46). To determine whether comparable consequences would occur in our *Dnmt1^tet/tet^* ES cell reprogramming model, we quantitatively assessed the methylation status on gDMDs of well characterized imprinted genes (*H19, Plag1, Gnas, Peg13, Mest* and *Kcnq1*). Subsequent to the suppression of DNMT1, imprinted genes underwent a substantial loss of DNA methylation, and following restoration of DNMT1 expression, all imprinted gDMDs failed to retrieve their original methylation levels (Figure 1D and E, *P* < 0.005). This methylation deficiency extended across several CpGs within the gDMDs (Figure 1E). Thus, the temporary disruption of methylation maintenance in the ES cell model leads to the permanent loss of heritable CpG methylation on gDMDs of imprinted genes, similar to the loss observed in embryos lacking complete DNMT1 function (6,9).

### Heritable DNA methylation exists outside the gDMDs of known imprinted genes

**Figure 2.**
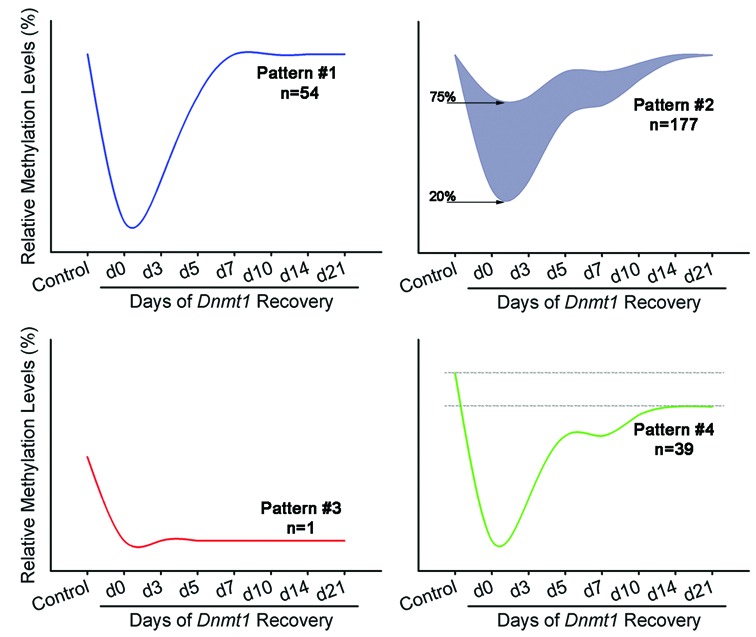
Temporal changes in DNA methylation following the depletion and reactivation of *Dnmt1* in *Dnmt1^tet/tet^* ES cells. RLGS analysis revealed that the transient suppression of *Dnmt1* generated four distinct methylation patterns for single copy loci *in Dnmt1^tet/tet^* ES cells. For pattern #1 (full de- and re-methylation: *n* = 54) loci, methylation was very low at d0 but returned to original levels by recovery d21. For pattern #2 (partial demethylation/full remethylation, *n* = 177) loci, following complete depletion of *Dnmt1*, methylation levels remained between 20% and 75% at d0 and then recovered original levels at d21. For pattern #3 (imprints: *n* = 1) locus, methylation was completely lost by d0 and no increase in methylation levels was observed during the full 21-day recovery period. For pattern #4 (loss of methylation: *n* = 39) loci, methylation levels were lost at d0 and were not completely reestablished by d21. ‘‘*n*’’ is the number of loci with specific pattern. See also Supplementary Figure S3 and Table S1.

Given that *Dnmt1^tet/tet^* ES cells can replicate the embryonic methylation reprogramming wave, we set out to determine whether heritable regions prone to DNA methylation maintenance failure resided outside gDMDs of imprinted genes. A 2D electrophoresis system (Restriction Landmark Genome Scanning; RLGS) exploiting methylation-sensitive or insensitive restriction endonucleases was used to establish a comprehensive timeline of DNA methylation profiles from control non-Dox treated *Dnmt1^tet/tet^* ES cells, and cells collected throughout the recovery period (d0, d3, d5, d7, d10, d14 and d21). We detected 290 single-copy loci (out of ∼2500) that displayed a decrease in methylation level following loss of DNMT1 (Supplementary Table S1). Re-expression of DNMT1 in the *Dnmt1^tet/tet^* ES cell line influenced the methylation recovery of the majority of affected loci in four major main patterns (Figure 2). For pattern #1 (full de- and re-methylation, *n* = 54) and pattern #2 (partial demethylation/full remethylation, *n* = 177), loci were able to completely recover their original methylation levels by d21. Some regions (pattern #2) were able to maintain partial DNA methylation (20–75%) during the period devoid of DNMT1 activity. For pattern #3 (imprinted, *n* = 1) and pattern #4 (>20% loss of methylation, *n* = 39), loci did not return to original levels by d21. With the specific combination of enzymes used, only one locus associated with an imprinted gene (*Zrsr1*) was apparent on mouse RLGS profiles, and it revealed a complete loss of methylation at d21 (pattern #3). The identity of only a limited number of the RLGS loci could be determined and the methylation of a few were quantitatively confirmed by pyrosequencing analysis (Supplementary Figure S3). The identification of pattern #4 loci suggested that heritable DNA methylation marks exist outside gDMDs of imprinted genes and require maintenance by DNMT1.

**Figure 3.**
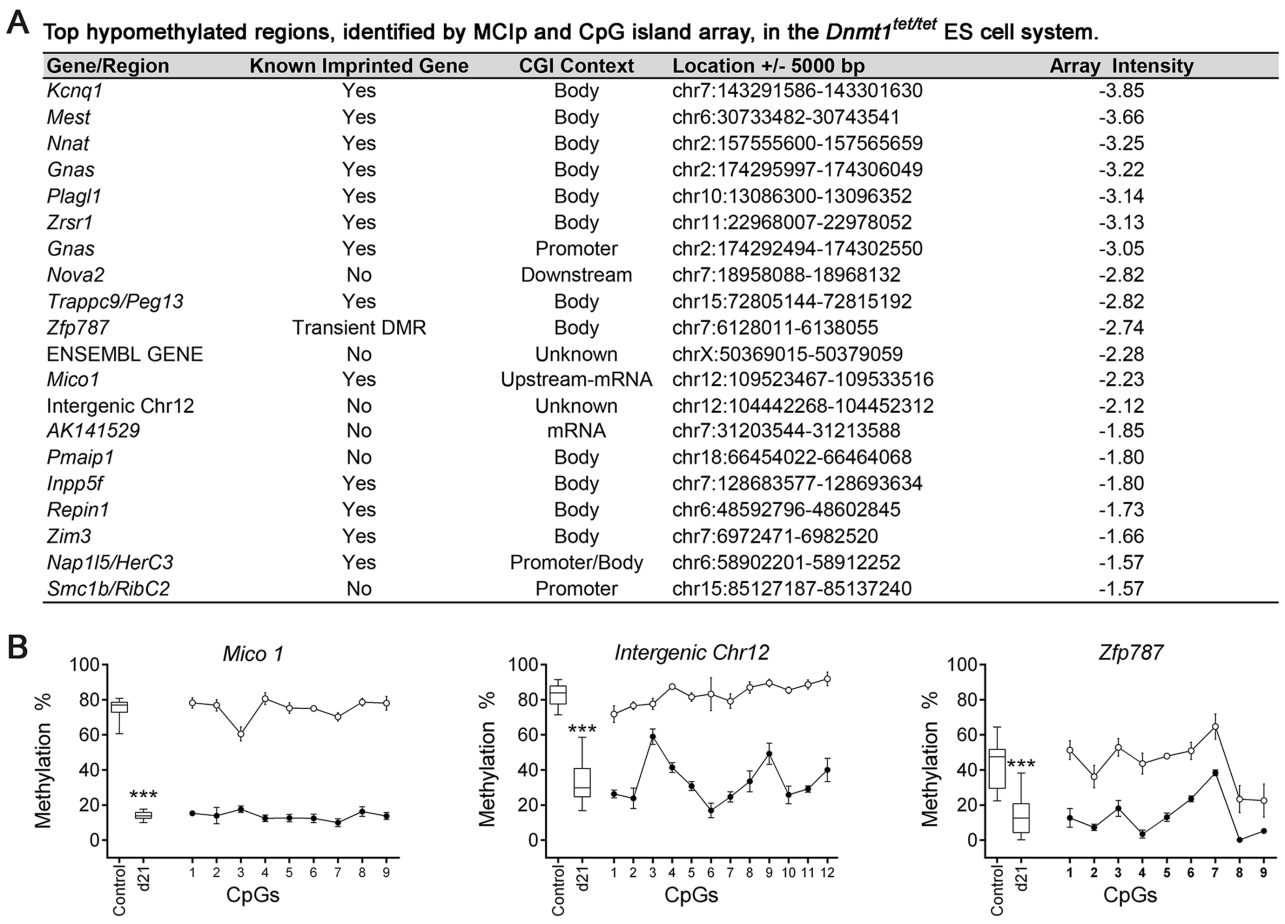
Regions identified by a combination of MCIp enrichment and array hybridization revealing loss of DNA methylation following the inactivation and reactivation of *Dnmt1* in the *Dnmt1^tet/tet^* ES cells. (**A**) Characteristics of top array probes revealing loss of methylation at d21 versus control *Dnmt1^tet/tet^* ES cells. (**B**) Hypomethylated regions quantitatively analyzed for CpG methylation levels. Methylation levels are shown as averages ± SD, Ctrl (*n* = 5) versus d21 (*n* = 6). Levels of methylation at single CpGs were quantified by Sequenom MassArray. Control versus d21 *Dnmt1^tet/tet^* ES cells: ^***^P < 0.005. See also Supplementary Figure S4 and Table S2.

To further investigate if specific regions outside gDMDs possessed heritable DNA methylation marks, we applied a combination of methyl-CpG Immunoprecipitation (MCIp) and mouse CpG island microarrays. DNA from control and d21 *Dnmt1^tet/tet^* ES cells was used for comparison and yielded a list of 725 CGI regions with reduced methylation following DNMT1 recovery (Figure 3A, Supplementary Table S2). Out of the 47 imprinted genes represented on this microarray, a total of 12 were identified among the top 2% probe cutoff used in our MCIP hypomethylation analysis. Decreases in methylation in the recovered *Dnmt1^tet/tet^* ES cells were detected across all chromosomes, except for the Y chromosome which was not represented on the CGI array used (Supplementary Figure S4). Regions revealing the most divergence in methylation were all associated with imprinted genes, highlighting the efficiency of our strategy in revealing hereditary methylation marks. Sequenom MassArray was used to validate the methylation of other regions in the same range of array intensity as imprinted genes, including *Mico1*, an imprinted non-coding RNA (47), *Zfp787*, a transient DMR (48) and an intergenic region of Chr12 (Figure 3B). These observations provide further evidence that improper maintenance of DNA methylation in the ES cell methylation reprogramming model leads to loss of inherited methylation outside known imprinted gDMDs.

### High resolution genome-wide assessment of novel heritable gDMD-like methylation

**Figure 4.**
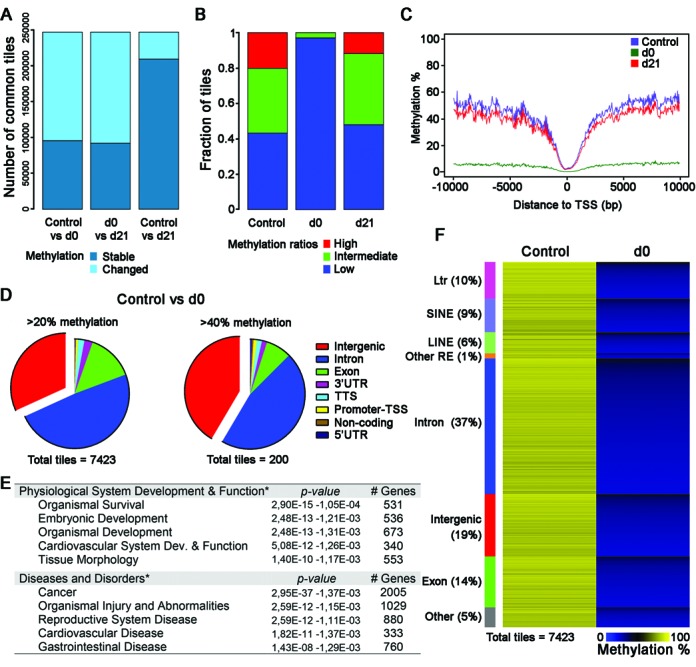
Global DNA methylation dynamics in ES cells subsequent to interruption and reactivation of *Dnmt1* in *Dnmt1^tet/tet^* ES cells. Paired end sequenced RRBS libraries of control (*n* = 3), d0 (*n* = 3) and d21 (*n* = 4). (**A**) Pairwise comparison of 100 bp tiles that revealed unchanged (stable) and changed methylation levels during inactivation of *Dnmt1* (control versus d0), recovery of *Dnmt1* (d0 versus d21) and subsequent to transient suppression of *Dnmt1* (control versus d21). Methylation variance cut offs of ≥20% and *T*-test *P* < 0.05. (**B**) Fraction of 100 bp tiles with high (>80%), intermediate (20–80%) and low methylation (<20%) values in ES cells with active (control), inactive (d0) and recovered (d21) *Dnmt1* expression. (**C**) Methylation mean surrounding the transcription start site (TSS) for all tiles in each treatment group. (D–F) Attributes associated with the differentially methylated regions observed between control and d0 *Dnmt1^tet/tet^* ES cells. These regions conserved considerable DNA methylation levels in the absence of DNMT1. (**D**) Representation of tile features that revealed a loss of at least 20% methylation at d0 compared to control, but still retained a portion larger than 20% (*n* = 7423) or 40% (*n* = 200) methylation of original levels. (**E**) Summary of biological functions associated to the genic related tiles that conserved at least 20% and 40% methylation of original levels at d0. Unique gene identifications were used for biological function associations. (**F**) Heat map representation of methylation levels among the 7423 tiles. Tiles were first clustered for presence of repeat elements, tiles without these classes were clustered for genic location in the genome, then were sorted for methylation levels at d0 within each cluster. Sequencing data use in (A)–(F) included all 100 bp tiles with >20-fold coverage. See also Supplementary Figures S5–S9 and Table S3.

To identify novel heritable methylation marks at a genome-wide level, we carried out a high resolution qualitative and quantitative examination of DNA methylation dynamics in the *Dnmt1^tet/tet^* ES cells using Reduced Representation Bisulfite Sequencing (RRBS). We first determined the methylation levels following the inactivation (control versus d0) and transient suppression of *Dnmt1* (control versus d21) (Figure 4). A substantial number of tiles (∼150 000) with altered DNA methylation were observed following the inactivation phase (Figure 4A), caused by the extensive increase in the tile fraction exhibiting low methylation (<20%) (Figure 4B). These ∼150 000 (Supplementary Figure S5) are distributed in 78 755 unique consecutive regions separated by a maximum of 500 bp (Supplementary Figure S6). Although low methylation (<20%) is observed, a total of 7423 tiles with intermediate methylation (20–80%) were still detectable (Figure 4D–F, Supplementary Table S3). Most of these intermediate methylated tiles (*n* = 5063; 68%) were located in genic regions (5′UTR, promoter-TSS, exon, intron, TTS, 3’UTR and non-coding) and were associated with various biological functions (Figure 4E). Some of these tiles (*n* = 200) retained substantial methylation levels, ranging from 40% to 74%. A significant enrichment in tile proportion was observed for intergenic and intron features for both categories of retained methylation (Supplementary Figure S7). For the 7423 intermediate-methylated tile collection, 26% of the loci (*n* = 1961) overlapped REs, and the majority (*n* = 5462; 74%) that did not overlap REs were within 1000 bp of a RE (Figure 4F, Supplementary Figure S8). This analysis shows that following the inactivation of DNMT1, genomic sequences other than REs were able to retain a significant portion of their methylation without DNMT1.

**Figure 5.**
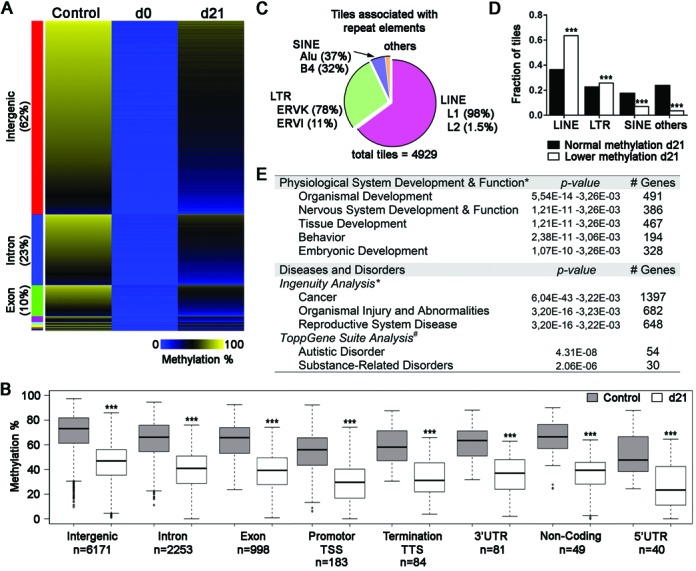
Suppression followed by reactivation of *Dnmt1* reveals specific functional classes affected and methylation deficits similar to imprinted genes. Genomic regions that exhibited altered methylation levels subsequent to the interruption and reactivation of *Dnmt1 in Dnmt1^tet/tet^* ES cells. (**A**) Heatmap representation of methylation levels for 9884 tiles that lost methylation at d0 and did not recover their original levels by day 21 (methylation variance >20% and *P* < 0.05). Methylation levels were clustered by genomic regions and sorted by intensity displayed in control *Dnmt1^tet/tet^* ES cells within each cluster. (**B**) Distribution of annotated sequence features presented in (A). ^***^P < 0.001 between control and d21 of same class, by Wilcoxon test. (**C**) Proportion of hypomethylated tiles at d21 associated with repeat elements (*n* = 4929). (**D**) Repeat element class distribution in tiles with normal methylation at d21 and tiles with loss (lower) of methylation at d21. ^***^P < 0.001 between normal and control of same class, by *χ*2-test. (**E**) Summary of biological functions associated with the genic related tiles that showed altered methylation levels at day 21. Out 3688 genic region tiles, 2353 coded for unique gene identifications and *2313 qualified for biological function by IPA software, and ^#^1964 qualified for biological function by ToppGene Suite software. See also Supplementary Figures S10–S14 and Table S4.

Next, we investigated how tiles responded to the demethylation and remethylation wave generated by the transient suppression of DNMT1 (control versus d21). As had been found with the RLGS and MCIp approaches, a fraction of tiles revealed altered methylation levels (Figure 4A) and a reduction in the total amount of highly methylated tiles was also observed (Figure 4B). This trait was recurrent between d21 samples and generated high correlation in hierarchical clusterings (Supplementary Figure S9). Distribution of mean DNA methylation levels specifically located around the TSS and flanking regions revealed a general state of genome-wide DNA methylation reduction following the transient suppression of DNMT1 (Figure 4C). A total of 10 069 tiles showed altered methylation following the transient suppression of DNMT1 (MethylKit analysis), with 9884 tiles revealing reduced levels (Figure 5A and B, Supplementary Table S4). Of these 9884 tiles, 262 overlapped with hypomethylated CGI regions found using the MCIP-based approach. We also observed 185 tiles (out of 10 069) displaying a methylation increase during the transient suppression of DNMT1 (discussed in supplemental results section). Tiles with methylation loss were distributed across all chromosomes (Supplementary Figure S10) with no correlation to established imprinted gDMDs. The only non-gDMD hypomethylated tiles found in proximity (<50 000 bp) to known gDMDs were associated to the promoter of *Aurkc* (5’ of *Zim3*) and a 5’ region of *Ctsh* (3’ of *Rasgrf1*). Loss of methylation in intergenic regions was significantly enriched compared to the other features (Supplementary Figure S11). The proportion of tiles (*n* = 3688, 37.3%) that mapped to genic regions (Figure 5B) related to normal physiological processes including development (organismal, nervous system, tissue and embryonic) and behavior (Figure 5E), as well as disorders such as cancer, organismal injury, reproductive system disease and autistic disorder. Analysis of the sequences associated to tiles with loss of methylation (*n* = 9884) revealed a significant enrichment of binding and transcriptional factor-motifs, such as *Elk4* involved in both transcriptional activation and repression (Supplementary Figure S12). Approximately half of the hypomethylated tiles (*n* = 4929) also overlapped with REs, with the majority being LINE elements. Tiles with lower methylation at d21 had a significantly higher proportion of LINE features compared to d21 tiles with normal methylation (DNA methylation similar to control). A total of 4955 tiles were not overlapping a RE but were within 1000 bp of the genomic structures (Supplementary Figure S13). Only 230 tiles were located in CGIs (Supplementary Figure S14), with most of these CGI tiles located in genic regions (84%, *n* = 197) with biological functions associated with development and cancer (Supplementary Figure S14C). The RRBS studies thus revealed that the methylation of numerous tiles associated with non-imprinted gDMD regions were perturbed following the transient suppression of DNMT1 and suggest that they could have heritable gDMD-like behaviors essential for developmental health.

### Loss of heritable non-DMD CpG methylation in embryos and placentae

**Figure 6.**
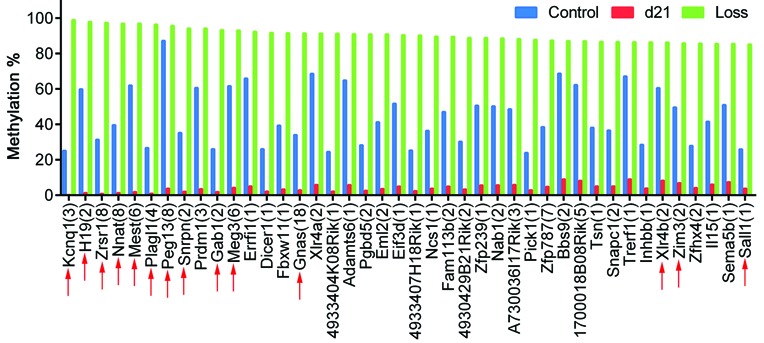
Classification by methylation reduction of annotated tiles in control versus d21 *Dnmt1^tet/tet^* ES cells. Top 42 candidates showing a methylation loss ranging from 100% to 85% at d21. Bars represent the average of methylation averages of tiles associated to unique gene IDs. The number of annotated tiles for each gene ID is indicated in brackets. Known mouse imprinted genes are depicted by red arrows. See also Supplementary Figure S15.

**Figure 7.**
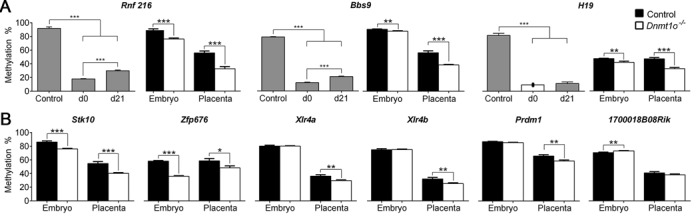
Heritable candidates revealing methylation loss in embryos and placentae lacking methylation maintenance during preimplantation. (**A**) Genomic regions that lost methylation following the temporary suppression of *Dnmt1* (gray bars: control, d0 and d21) in *Dnmt1^tet/tet^* ES cells were examined in 9.5dpc embryos and placentae from control and *Dnmt1^Δ1o/Δ1o^* mothers (**A** and **B**). Quantified by Pyrosequencing. ^***^P < 0.005, ^**^P < 0.01, *P < 0.05. See also Supplementary Figures S16–S18.

To compare gDMDs to non-imprinted regions with perturbed methylation following transient DNMT1 loss, annotated tiles were classified by the percentage of total methylation loss. Regions with the most methylation loss were confirmed imprinted genes (total imprinted genes, *n* = 15) (Figure 6 and Supplementary Figure S15, 100–80% methylation loss). However, many other non-imprinted associated regions (*n* = 75) showed similar levels of methylation loss, suggesting heritable attributes. The same approach applied to CGI-tiles gave similar results, with a very few non-imprinted annotated sequences showing total methylation loss comparable to that of imprinted genes (Supplementary Figure S14D). Pyrosequencing quantification of a subset of regions/tiles (*Bbs9, Rnf216, Xlr4a, Xlr4b, 1700018B08Rik, Snrpn* and *H19*) confirmed that d21 methylation was significantly lower than the original levels observed in control *Dnmt1^tet/tet^* ES cells (Figure 7, Supplementary Figure S16). Compared to an imprinted gDMD such as *Meg3, Snrpn* and *H19*, all tested regions revealed a partial recovery of methylation by d21. These sequences are potentially novel developmental marks with imprinted-like behaviors that require continued or uninterrupted DNMT1 activity during each early embryonic division in order to be accurately inherited.

To test whether the novel regions identified in the ES cell model behave like the gDMDs of imprinted genes in the *in vivo* context, we used the *Dnmt1^Δ1o/Δ1o^* mouse model, in which absence of maternally derived DNMT1o at the eight-cell embryonic stage causes failure in the proper maintenance of imprinted gDMD methylation. Lack of DNMT1o leads to reduction (∼25–50%) of DNA methylation on imprinted gDMDs of mid-gestation embryos and placentae, as well as to phenotypic abnormalities and lethality (13,15,16,49). Embryos and placentae were collected at 9.5dpc and the methylation status assessed for 11 heritable candidates. For *Rnf216, Bbs9, Stk10 and Zfp676*, significant reduction in methylation levels was observed in embryos and placentae from *Dnmt1^Δ1o/Δ1o^* females, similar to the expected average loss observed for the imprinted gene *H19* (Figure 7). *Xlr4a, Xlr4b* and *Prdm1* showed significant decreases in placentae from *Dnmt1^Δ1o/Δ1o^* females, whereas *1700018B08Rik, Eml2, Zfhx4* and *Zfp239* revealed slight but significant methylation increase in embryos from *Dnmt1^Δ1o/Δ1o^* females (Supplementary Figure S16). Decreased methylation was variable in individual samples (Supplementary Figures S16–S18), a typical trait associated with the mosaic nature of the *Dnmt1^Δ1o/Δ1o^* model (13). Although imprinted *Xlr4b* methylation was altered in placentae from *Dnmt1^Δ1o/Δ1o^* females, no changes were observed in the related embryos, however, the region tested could be outside the actual gDMD as it remains to be defined (50). These results demonstrate that improper methylation maintenance during the embryonic reprogramming period causes DNA methylation loss on sequences outside of known imprinted gDMDs.

## DISCUSSION

### Modeling genomic methylation and demethylation

It is well accepted that steady-state levels of genomic methylation are maintained largely through the action of DNMT1, the workhorse cytosine maintenance methyltransferase in mammalian cells. Changes in patterns of genomic methylation, which occur commonly during early embryonic stages and virtually all stages of female and male gametogenesis, however must require *de novo* methylation and demethylation processes. These processes can affect different DNA sequences at different times, and their dependence on DNMT1 is not fully understood and even controversial. For example, genomic methylation is completely or nearly completely lost during the generation of primordial germ cells, yet is it not clear whether this demethylation is due to passive demethylation (loss of DNMT1 activity) or active demethylation or a combination of both. Similarly, during preimplantation, the majority of genomic methylation is lost, again by poorly understood passive and/or active demethylation processes, whereas gDMD methylation is concomitantly maintained by DNMT1 activity. Furthermore, little detailed information about the maintenance requirement of specific CpG methylation patterns outside imprinted regions has been generated from preimplantation embryos. Despite the loss of the majority of genomic methylation during preimplantation, much of the genome is remethylated following implantation into the uterus. The demethylation and remethylation of the *Dnmt1^tet/tet^* ES-cell genome, induced by administration and removal of doxycycline, mimic many of the profound developmental methylation changes and have allowed us to better understand the roles of DNMT1 in these processes.

Modulating the complete and transient suppression of DNMT1 in *Dnmt1^tet/tet^* ES cells not only recreated the characteristic demethylation and remethylation wave, but as anticipated, caused the loss all heritable imprinted gDMD methyation. The transient suppression of DNMT1 is equivalent to a complete embryonic deficiency of DNMT1 function (8,46). Reactivation of DNMT1 in *Dnmt1^tet/tet^* ES cells re-established DNA methylation profiles on most genomic sequences, but not on imprinted gDMDs, since methylation of these sequences is only established in a parent-of-origin manner during gametogenesis by DNMT3a/3b. The transient suppression of DNMT1 in our reprogramming model also recreated the unique and characteristic bimodal methylation behaviors observed on various REs during preimplantation (29,44,45). Following the inactivation of DNMT1, elements such as LINE-1 showed nearly full demethylation whereas others such as SINE only partially lost their methylation, similar to previous observations in preimplantation embryos.

### Rapid changes in genomic methylation following transient DNMT1 loss

One real advantage of the *Dnmt1^tet/tet^* cells is the strict pharmacologic control of intracellular DNMT1 enzyme levels. This has revealed a number of distinct features accompanying the inactivation of *Dnmt1* gene transcription and consequential rapid loss of the DNMT1 protein. Virtually all sequences lose methylation, yet the extent of methylation loss is unevenly distributed across the genome. To better understand the dynamics of inherited CpG methylation patterns during the reprogramming phase, we generated large scale and complementary DNA methylation analyses in our DNA methylation reprogramming ES cell model. As expected, inactivation of DNMT1 activity yielded a dramatic drop in genomic DNA methylation levels, with imprinted gDMD sequences losing essentially all methylation. Conversely, REs retained a portion of their marks, but surprisingly they only represented 26% of regions with retained methylation, the rest being mainly found in gene-associated regions distributed amongst all chromosomes. The residual methylation outside gDMD sequences must be due to *de novo* methyltransferase activities, presumably acting on replicated DNA. This notion is consistent with the requirement of a DNMT3 *de novo* methyltransferase for long-term, sustained genomic methylation in ES cells (17,19). In contrast, unmethylated alleles of known gDMD sequences appear to be immune to these *de novo* methyltransferase activities. The mechanism of this immunity is unknown, yet clearly a cardinal feature of gDMDs.

Upon re-expression of DNMT1 in *Dnmt1^tet/tet^* cells, induced by complete removal of doxycycline from the cell-culture medium, remethylation of the genome occurs rapidly. Interestingly, a partially (*de novo*) methylated state may be a requirement for rapid DNMT1-mediated remethylation, as completely unmethylated gDMDs sequences are immune to this remethylation (19,51). Because partially methylated sequences in the absence of DNMT1 likely undergo repetitive *de novo* methylation with each cell cycle, there may be a mechanistic connection between one or more DNMT3 enzyme and DNMT1 during this process. Whether the remethylation of many genomic sequences during early embryogenesis is mediated by a similar or identical mechanism is not clear.

### gDMD and gDMD-like sequences

In at least two ways, gDMD and gDMD-like sequences are different from the bulk of genomic sequences. First, they show an absolute dependence on DNMT1 to maintain methylation. Second, they require stage-specific *de novo* methyltransferase activity to increase their methylation from the unmethylated ground state, activity not found in *Dnmt1^tet/tet^* ES cells. How DNA methyltransferase enzymes are able to discriminate among genomic loci, to methylate some but not all, is still poorly understood. Interestingly in our ES cell reprogramming model, immunity to DNMT1 remethylation included many sequences outside known gDMDs. Half of these sequences overlapped REs, with the majority being LINE elements. Continuous and cooperative activity from *de novo* and maintenance methyltransferases are known to be needed for the methylation of LINEs, since in ES cells their methylation is ineffectively maintained single-handedly by DNMT1, or by DNMT3a/3b without DNMT1 (19).

Out of ∼10 000 tiles showing methylation reduction (>20%) following the transient inactivation of DNMT1 in *Dnmt1^tet/tet^* ES cells, only a small fraction of these exhibited a pronounced (>80%) reduction in methylation. These were associated with 90 genic sequences, of which 15 were known imprinted gDMDs and two (*Zfp787* and *Xlr4a*) had affiliations with imprinting and allelic immunity from methylation. *Zfp787* is a transient gDMD that acquires maternal allelic methylation in the oocyte, however following postimplantation *de novo* methylation both parental alleles display similar levels in 9.5dpc embryos (48). *Xlr4a* is associated with a cluster of X-linked genes with paternal-specific, imprinted transcriptional repression, independent of X-chromosome inactivation. In contrast to the imprinted genes from this cluster (*Xlr3b, Xlr4b* and *Xlr4c*), *Xlr4a* is expressed from both parental alleles and thus not imprinted (50). Interestingly, *Xlr4a* only showed partial loss of methylation in placentae from *Dnmt1^Δ1o/Δ1o^* females. Other presumably non-imprinted regions like *Rnf216, Bbs9, Stk10* and *Zfp676* revealed significant loss of methylation in both embryos and placentae derived from *Dnmt1^Δ1o/Δ1o^* females. These findings are consistent with the existence of non-imprinted sequences with epigenetic gDMD-like features that set them apart from the vast majority of genomic sequences. Furthermore, these sequences are associated with genes that are implicated in normal developmental processes, including nervous system function and behavior; the disruption of these genes is linked to a variety of abnormalities as well as to developmental and neurological disorders.

In conclusion, we describe an ES cell model that emulates the demethylation and remethylation dynamics of preimplantation development. One advantage of this model is that cell numbers are not limiting, allowing an in depth, robust and quantitative analysis of genome-wide and site-specific DNA methylation patterns. The model reproducibly replicated both the demethylation and remethylation dynamics of the bulk of sequences in the genome. In addition, the model also revealed the presence of novel heritable methylation marks, some of which were reduced in *in vivo* tests, signifying regions of potential important developmental relevance especially during the early stages of embryo development.

## SUPPLEMENTARY DATA

Supplementary Data are available at NAR Online.

SUPPLEMENTARY DATA
