# Supplementary material for: Transient DNMT1 suppression reveals hidden heritable marks in the genome
Source: Nucleic Acids Res. 2015 Jan 10;43(3):1485–97. doi: 10.1093/nar/gku1386 (PMC4330356; doi:10.1093/nar/gku1386)
Supplement: SUPPLEMENTARY DATA [file supp_43_3_1485__index.html]

Transient DNMT1 suppression reveals hidden heritable marks in the genome — Transient DNMT1 suppression reveals hidden heritable marks in the genome — SUPPLEMENTARY DATA 

# Transient DNMT1 suppression reveals hidden heritable marks in the genome

## SUPPLEMENTARY DATA

**Files in this Data Supplement:**

- SUPPLEMENTARY DATA
- SUPPLEMENTARY DATA
- SUPPLEMENTARY DATA
- SUPPLEMENTARY DATA
- SUPPLEMENTARY DATA
