# Supplementary material for: Transient DNMT1 suppression reveals hidden heritable marks in the genome
Source: Nucleic Acids Res. 2015 Jan 10;43(3):1485–97. doi: 10.1093/nar/gku1386 (PMC4330356; doi:10.1093/nar/gku1386)
Supplement: SUPPLEMENTARY DATA [file supp_gku1386_nar-01920-v-2014-File009.pdf]

Supporting Text - Results

Methylation gain following transient suppression of DNMT1

Unexpectedly, the transient suppression of DNMT1 in our ES cell reprogramming model generated a small number of regions with increased DNA methylation (Figure S19). This was first observed in our RLGS analysis where 19 single-copy loci revealed higher methylation levels in d21 *Dnmt1*$^{tet/tet}$ ES cells as compared to controls (Figure S19, Table S1; increased methylation). Loci with this behavior were regrouped and labeled pattern #5 (increased methylation). Similar observations were made in MCIp experiments where 70 probes displayed increased methylation (Figure S20, Table S2; increased methylation). Although significant, the methylation variations between control and d21 (Figure S20B) were not as considerable as those observed for genes with methylation loss at d21 (Figure 3B). High-resolution DNA methylation profiling by RRBS uncovered 185 tiles with increased methylation. Most of these tiles had low to median methylation levels in control *Dnmt1*$^{tet/tet}$ ES cells, levels were depleted by d0, and following the reactivation and recovery period of DNMT1 at d21 substantial methylation increases were observed (Figure S21, Table S4; increased methylation). Only 49 tiles were associated with genic regions (34 unique ID) and the top biological functions are summarized in Figure S21D. Interestingly, a total of 162 tiles overlapped repeat elements, with almost all of them being L1 elements. Pyrosequencing quantification of 2 regions/tiles that showed methylation increases, *Miip* and *Mllt4*, confirmed that d21 methylation was significantly higher than the original levels observed in control *Dnmt1*$^{tet/tet}$ ES cells ($p<0.005$) (Figure S22). In order to validate if this methylation increase could be observed in embryos with defective methylation maintenance during the embryonic reprogramming wave, we took advantage of the *Dnmt1*$^{\Delta 1o/\Delta 1o}$ mouse model (detailed in the main result text). DNA methylation was assessed in embryos and placentae (9.5dpc) and revealed no significant methylation increases for *Miip* or *Mllt4* (Figure S22). Interestingly, for *Mllt4,* a significant decrease in methylation was observed in embryos from the *Dnmt1o*-deficient mother. The methylation increase observed for these loci/regions might be an artifact created by our *Dnmt1*$^{tet/tet}$ ES cells. The significant methylation decrease created by the absence of *Dnmt1o* during a single embryonic stage suggests that some of these sequences might be sensitive to defects in the methylation maintenance machinery.

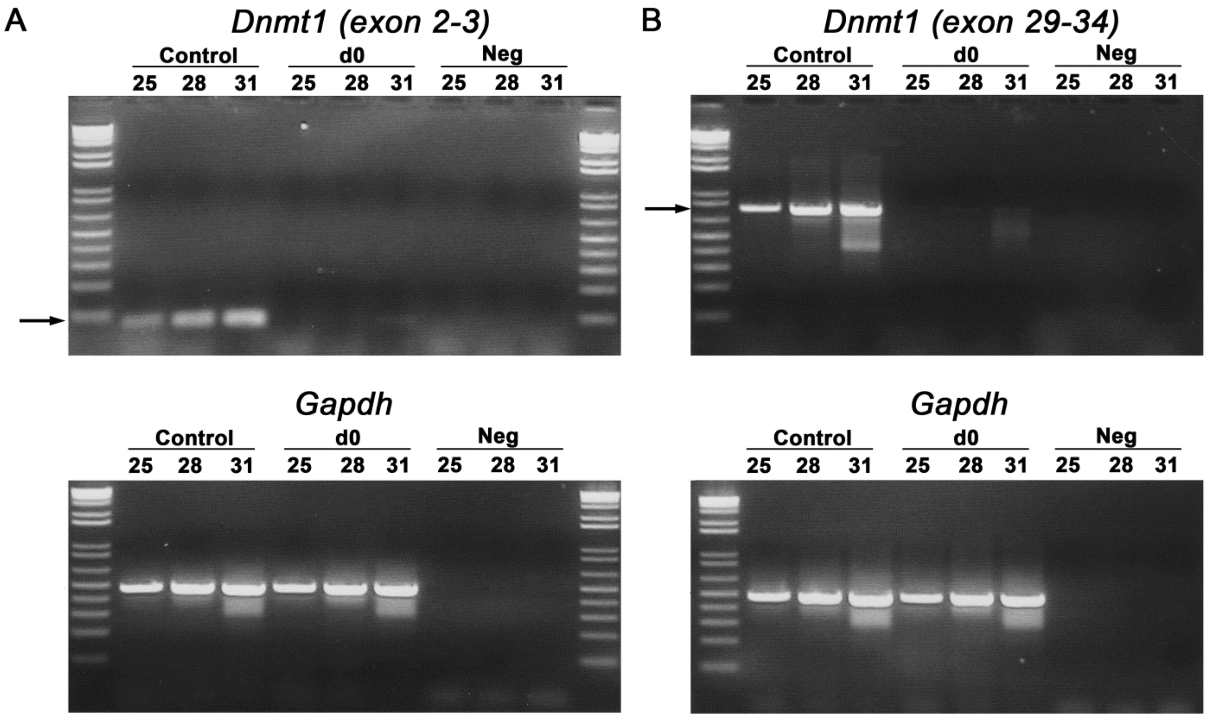

Figure S1, related to Figure 1. Expression of *Dnmt1* mRNA in *Dnmt1*$^{tet/tet}$ ES cells at d0 following Dox inactivation treatment. Expression of portions in the 5' and 3' ends of the *Dnmt1* mRNA in control *Dnmt1*$^{tet/tet}$ ES cells and following the 6 day Dox inactivation treatment (d0). *Gapdh* was used as the reference gene (500bp fragment). **A)** Expression of the 5' portion of *Dnmt1* mRNA (exon 2 and 3) is visible in control *Dnmt1*$^{tet/tet}$ ES cells whereas it is lost following Dox treatment at d0. Arrow indicates the 80bp fragment. **B)** Expression of the 3' portion of *Dnmt1* RNA (exon 29-34) is visible in control *Dnmt1*$^{tet/tet}$ ES cells whereas it is lost following Dox treatment at d0. Arrow indicates the 788bp fragment. 20ng of cDNA from cells were used for PCR. Cycle conditions: 94C 10min (94C 0.45sec, 58C 0.45sec, 72C 1min) 25/28/or 31 cycles.

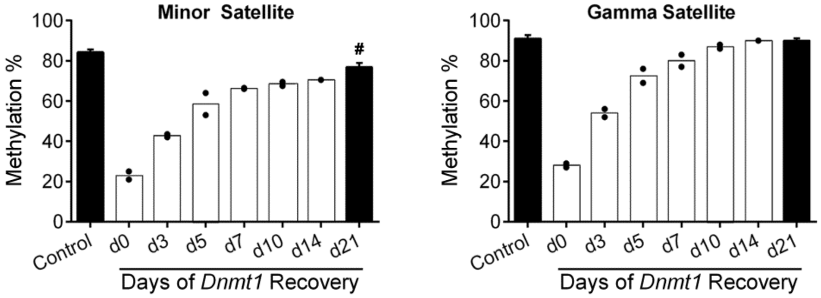

Figure S2, related to Figure 1. DNA methylation of repeat sequences following the interruption and reactivation of *Dnmt1* in *Dnmt1*$^{tet/tet}$ ES cells after 21 days. Methylation levels are shown as average methylation ± SD (Ctrl (n=5) vs d21 (n=6)). Methylation levels were also quantified from d0 to d14 (n=2) in order to visualize how methylation patterns were restored following the reactivation of *Dnmt1*. Repeat sequences (Minor and Gamma Satellite) were able to mostly or completely regain their original methylation levels following the reactivation of *Dnmt1*. Quantified by Sequenom MassArray. # p=0.058.

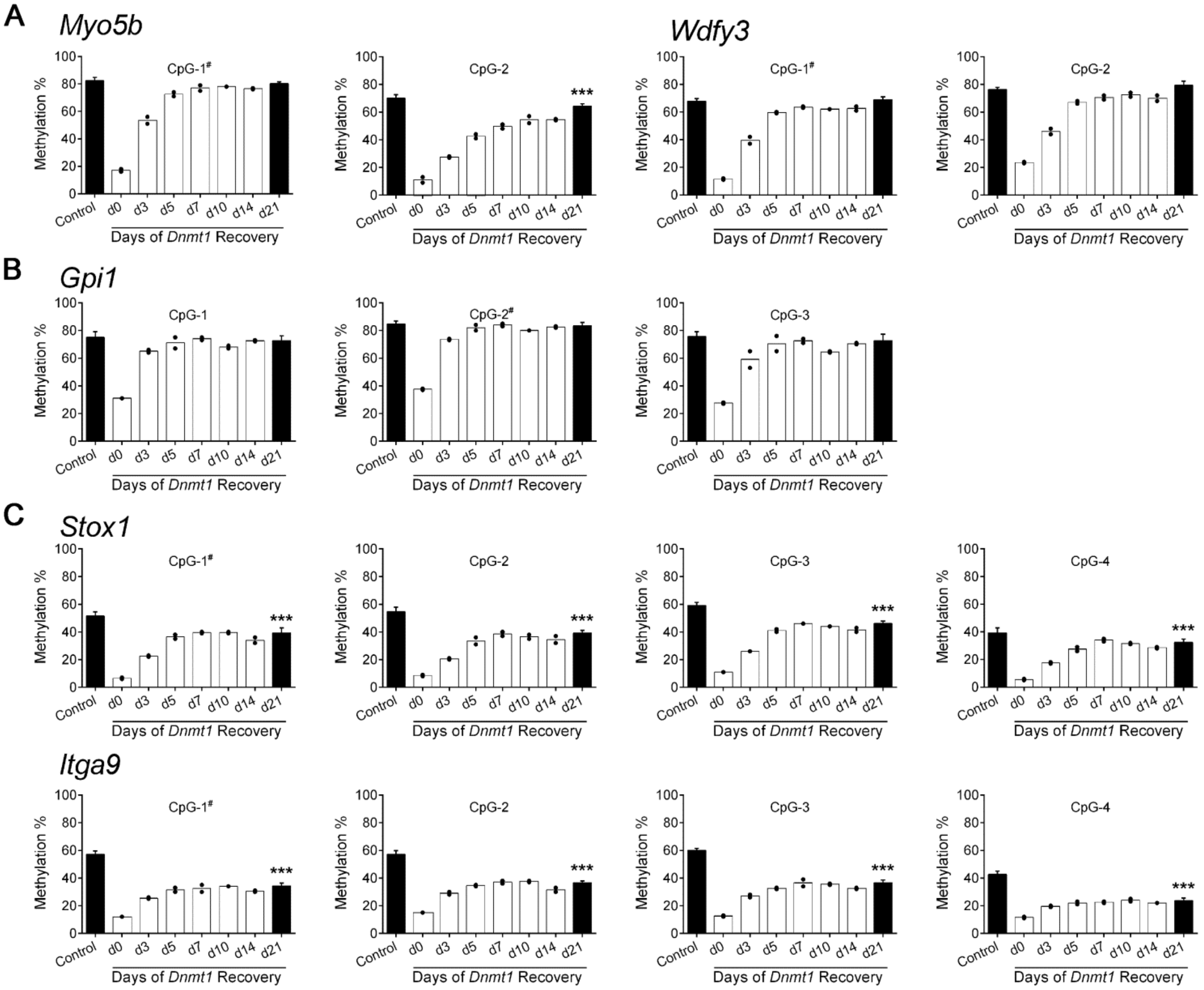

Figure S3, related to Figure 2. Quantitative validation of DNA methylation patterns for loci identified by RLGS. Methylation levels are shown as average methylation ± SD, Ctrl (n=5) vs d21 (n=6). Methylation levels were also quantified between d0 and d14 (n=2) to visualize how methylation patterns were restored following the reactivation of *Dnmt1*. The CpG in the *Not*I site examined by RLGS is denoted by #. Methylation status of neighboring CpGs was also quantified. **A)** Pattern #1, **B)** Pattern #2, and **C)** Pattern #4. Quantified by pyrosequencing. Control vs d21 *Dnmt1*$^{tet/tet}$ ES cells: *** p<0.005.

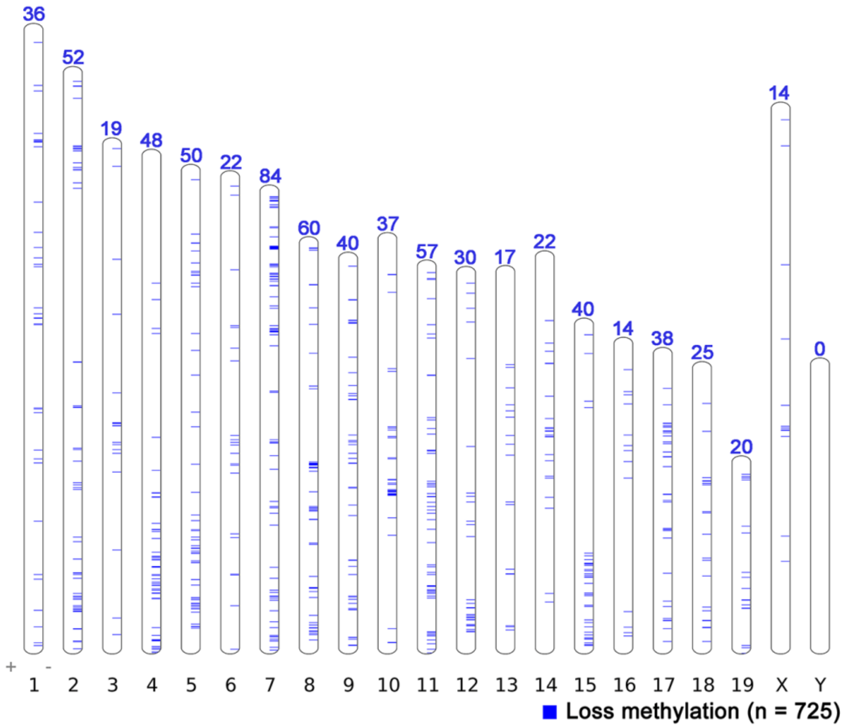

Figure S4, related to Figure 3. Chromosome distribution of the loci with loss of methylation following transient suppression of *Dnmt1*$^{tet/tet}$ in ES cells. Loci identified by a combination of Methyl-CpG Immunoprecipitation (MCIp) and mouse CpG island microarrays. Digits above chromosomes depict number of loci with loss of methylation. Control vs d21 *Dnmt1*$^{tet/tet}$ ES cells.

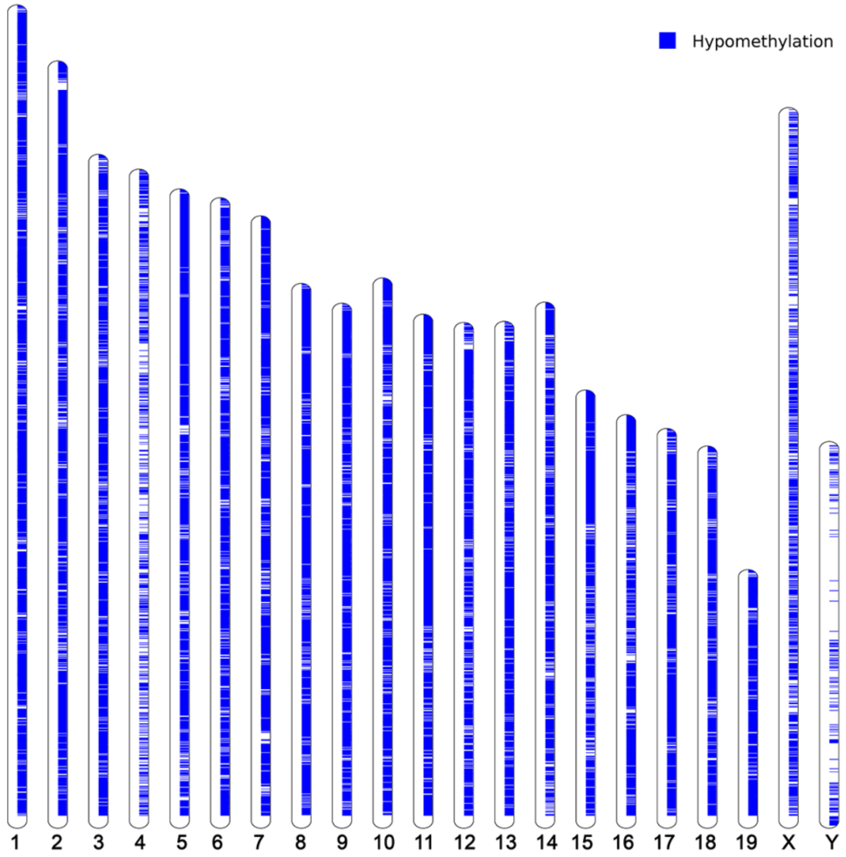

Figure S5, related to Figure 4. Chromosomal representation of loci with loss of DNA methylation at d0 following absence of methylation maintenance by *Dnmt1* in *Dnmt1*$^{tet/tet}$ ES cells. Blue marks represent 100bp tiles with altered DNA methylation between control and d0 *Dnmt1*$^{tet/tet}$ ES cells.

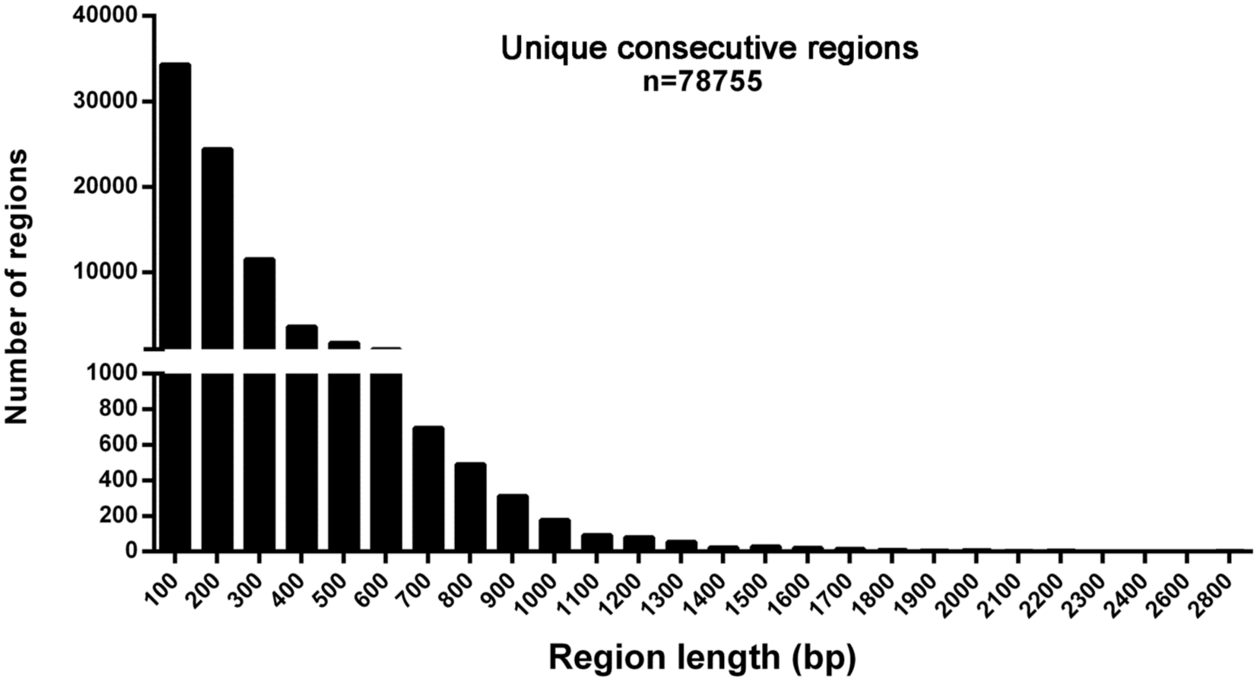

Figure S6, related to Figure 4. Unique regions showing consecutive/contiguous tiles separated by a maximum of 500bp associated with altered DNA methylation at d0 in *Dnmt1*$^{tet/tet}$ ES cells. From the ~150,000 different tiles a total of 78,755 regions were observed, ranging in size from 100bp (n=33,324 regions) to 2800bp (n=3 regions).

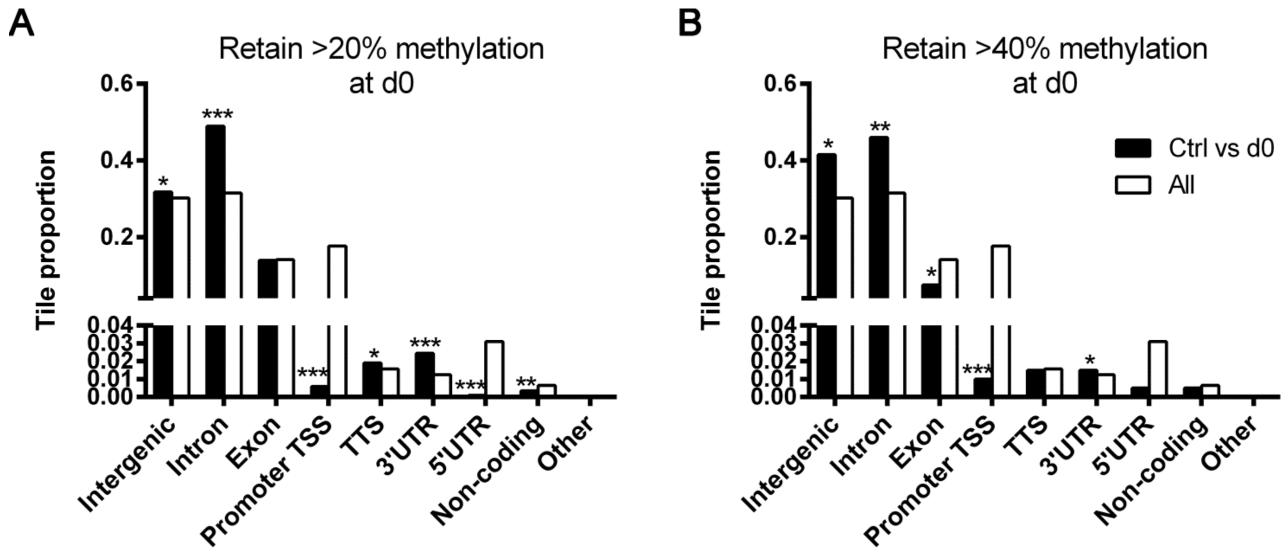

Figure S7, related to Figure 4. Enrichment analysis of genomic features. Analysis on tiles that lost greater than 20% methylation between control vs d0 *Dnmt1*$^{tet/tet}$ ES cells, but **A)** retained >20% and **B)** >40% methylation. *** $p<0.005$, ** $p<0.01$, * $p<0.05$ between tiles from Ctrl vs d0 and All tiles, by Chi-square.

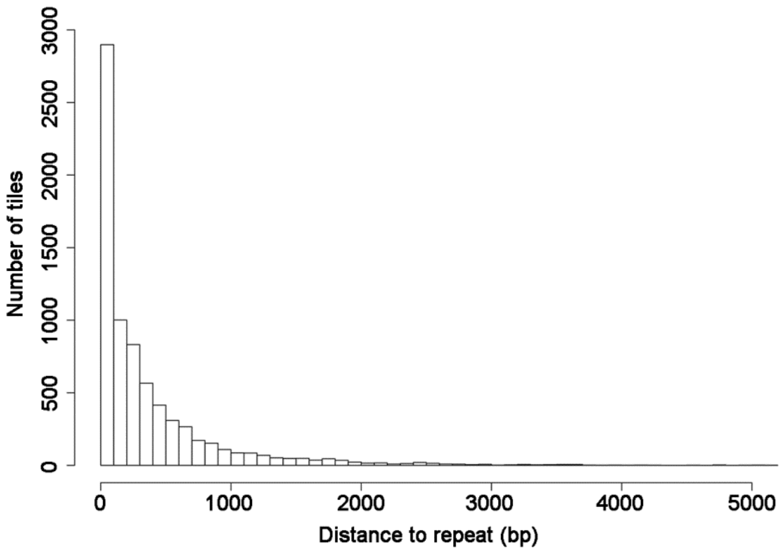

Figure S8, related to Figure 4. Proximity analysis to repeat elements of the 7,423 tiles that lost >20% methylation in control vs d0 *Dnmt1$^{tet/tet}$* ES cells but retained >20% methylation. In the 7,423 tiles, 1,961 were directly overlapping repeats and 937 were within 100bp (total of 2898 tiles represented in the first column).

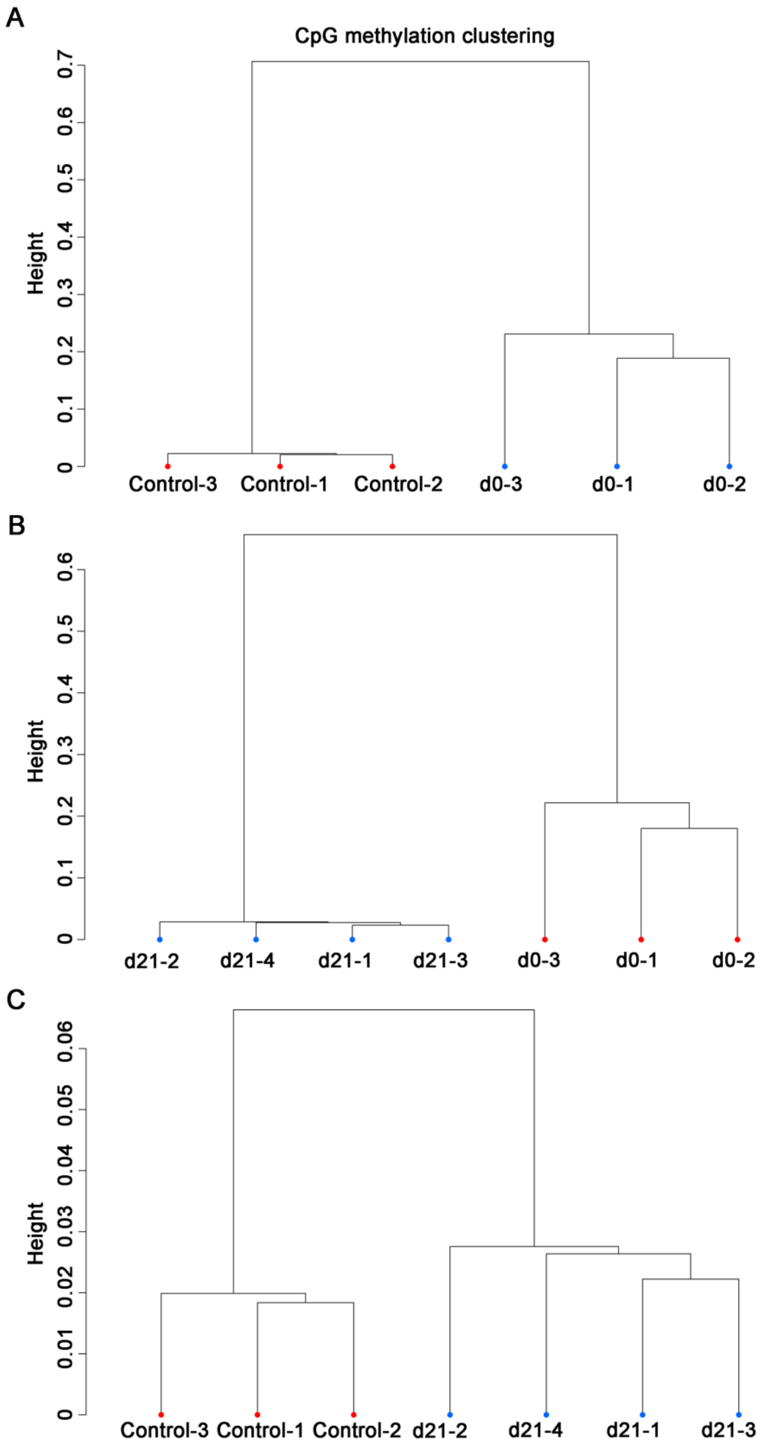

Figure S9, related to Figure 4. CpG methylation clustering diagram between samples. Distance method: "correlation", clustering method: "Ward". Samples analyzed, *Dnmt1*$^{tet/tet}$ ES cells : **A)** Control vs d0, **B)** d0 vs d21 and **C)** Control vs d21. Number following the *Dnmt1*$^{tet/tet}$ ES cell treatment represents the sample number.

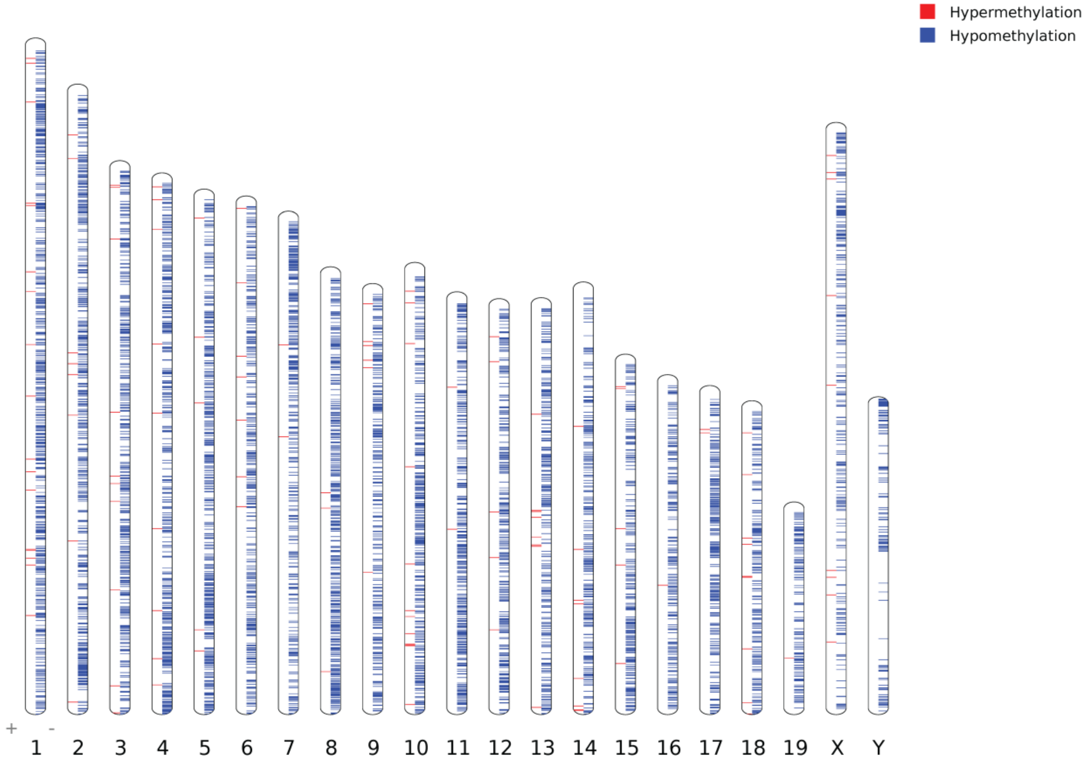

Figure S10, related to Figure 5. Chromosomal representation of loci with DNA methylation alterations following transient absence of DNA methylation maintenance by *Dnmt1* at d21 in *Dnmt1*$^{tet/tet}$ ES cells. Blue (hypomethylation) and red (hypermethylation) marks represent 100bp tiles with altered DNA methylation between control and d21 *Dnmt1*$^{tet/tet}$ ES cells.

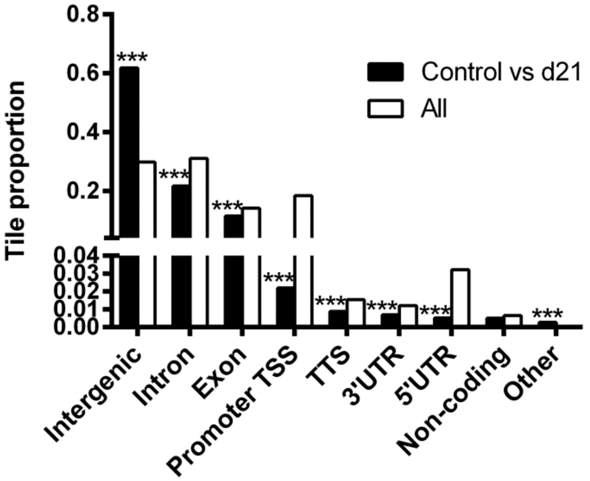

Figure S11, related to Figure 5. Enrichment analysis of genomic features. Comparison of the 9,884 tiles that lost methylation at d0 and did not recover their original levels by day 21 in *Dnmt1*$^{tet/tet}$ ES cells with all tiles sequenced. *** p<0.005 between tiles from Ctrl vs d21and All tiles, by Chi-square.

	log P-value	% of Targets	Best/Match Motif
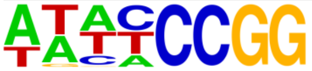	-3.8e+03	75.8%	Elk4
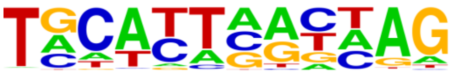	-2.5e+03	20.8%	Lhx6
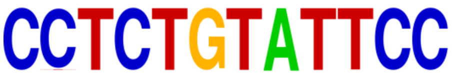	-2.4e+03	6.8%	Tead4
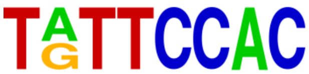	-2.4e+03	17.4%	Nfatc2
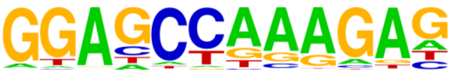	-2.1e+03	21.0%	Med-1
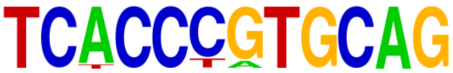	-2.0e+03	7.9%	Zbtb3
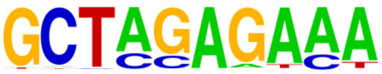	-1.8e+03	15.1%	Prdm1 /Bmi1
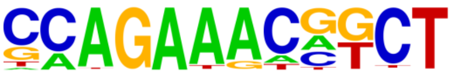	-1.7e+03	7.3%	ETS: E-box
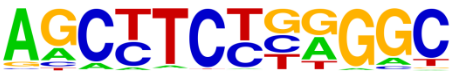	-1.7e+03	10.4%	Spi1
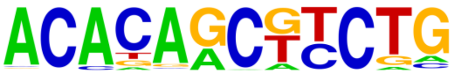	-1.7e+03	8.4%	Sox10

Figure S12, related to Figure 5. **Top *De novo* HOMER DNA binding motif analysis for tiles with methylation reduction in control vs d21 *Dnmt1*$^{tet/tet}$ ES cells**. DNA binding motifs were analyzed using HOMER on 100bp tiles (n=9,984) ± 100 bp

.

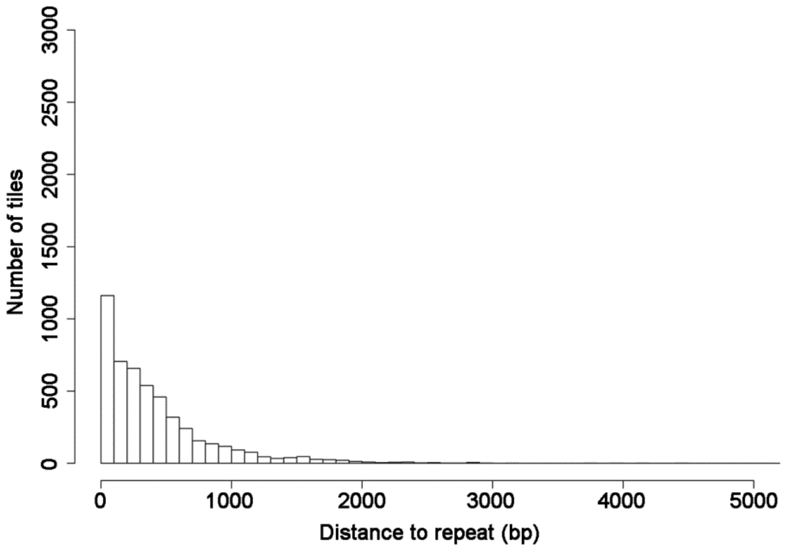

Figure S13, related to Figure 5. Proximity analysis to RE for non-RE associated sequences with methylation loss between control and d21 *Dnmt1*$^{tet/tet}$ ES cells. A total of 9,884 tiles lost methylation between control and d21 *Dnmt1*$^{tet/tet}$ ES cells, from these 4,955 were not overlapping a RE. Distance of these 4,955 tiles from RE structures are represented.

A

B

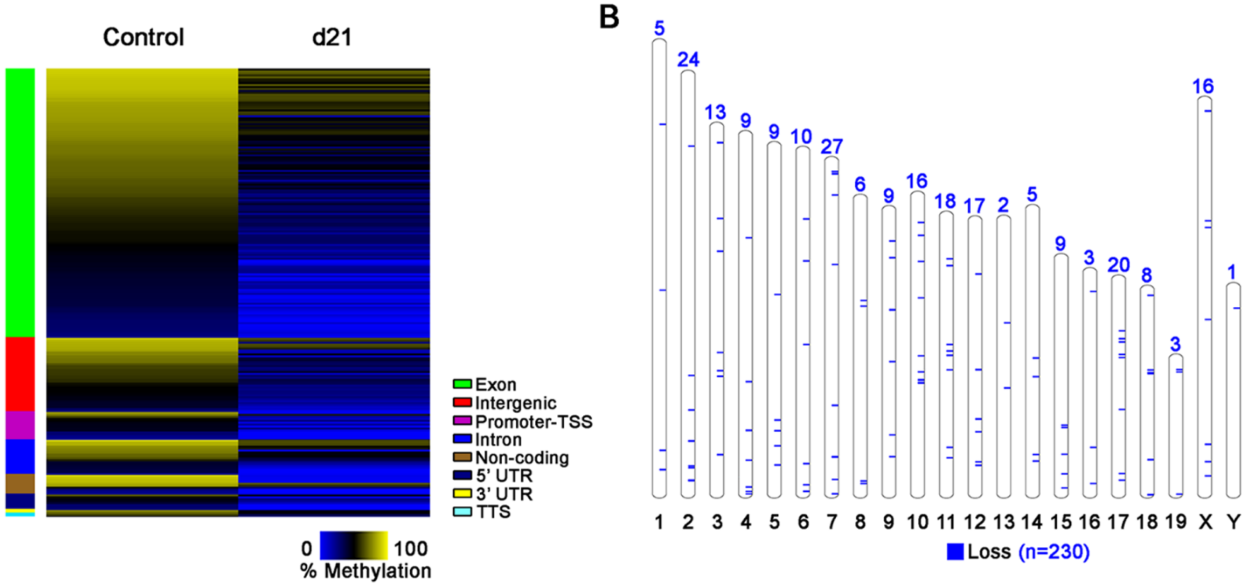

C

Biological function	# genes
100 unique gene ID qualified for analysis	
**Physiological system development and function**	
Embryonic Development	11
Connective Tissue Development & Function	10
Organ Development	8
Organ Morphology	8
**Diseases and disorders**	
Cancer	45
Developmental Disorder	15
Renal and Urological Disease	7

D

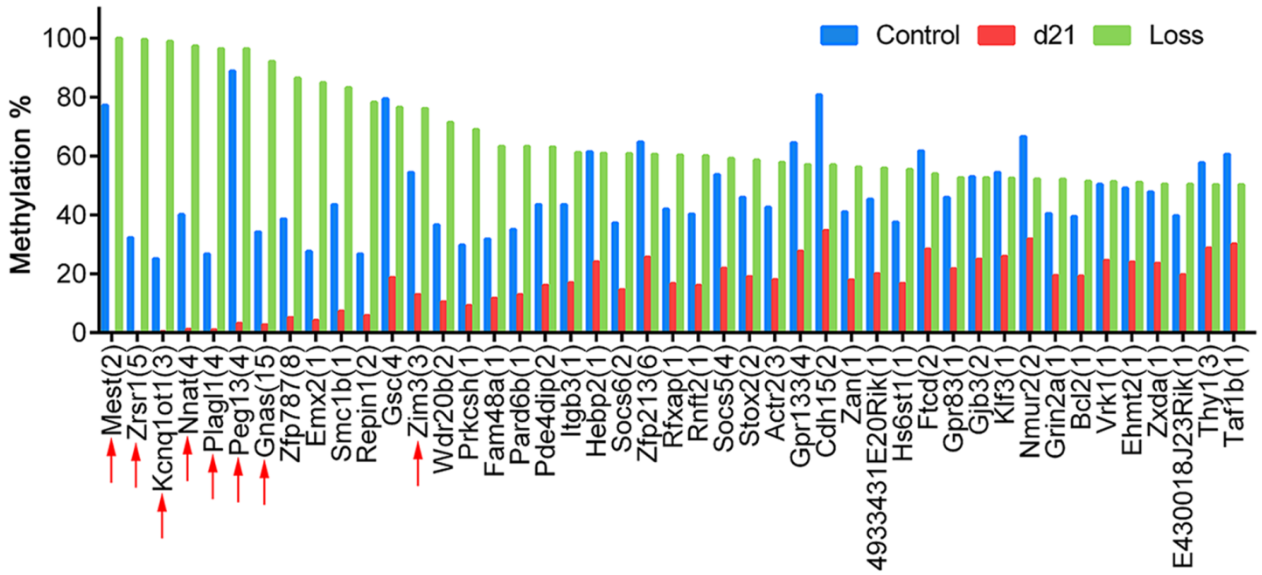

Figure S14, related to Figure 5. Genomic regions overlapping CpG islands with altered methylation levels following the interruption and reactivation of *Dnmt1*. A) Heatmap representation of methylation levels for 230 tiles with altered methylation levels at day 21 that overlapped CpG islands

(CGIs). Tiles were sorted by functional classes and clustered within each class by methylation intensity of control *Dnmt1$^{tet/tet}$* ES cells. **B)** Chromosome distribution of the 235 identified 100bp CGI-tiles with loss or gain of methylation at d21. Number of tiles with methylation loss (blue) are indicated above each chromosome. **C)** Summary of biological functions associated to the genic related CGI tiles that showed altered methylation levels at day 21. Unique gene identification was used for biological function association. **D)** Top 45 CGI-associated tile candidates showing a methylation loss ranging from 100% to 50% in control vs d21 *Dnmt1$^{tet/tet}$* ES cells. Bars represent the average of methylation averages of tiles associated to unique gene IDs. The number of tiles for each gene is indicated in brackets. Known mouse imprinted genes are depicted by red arrows.

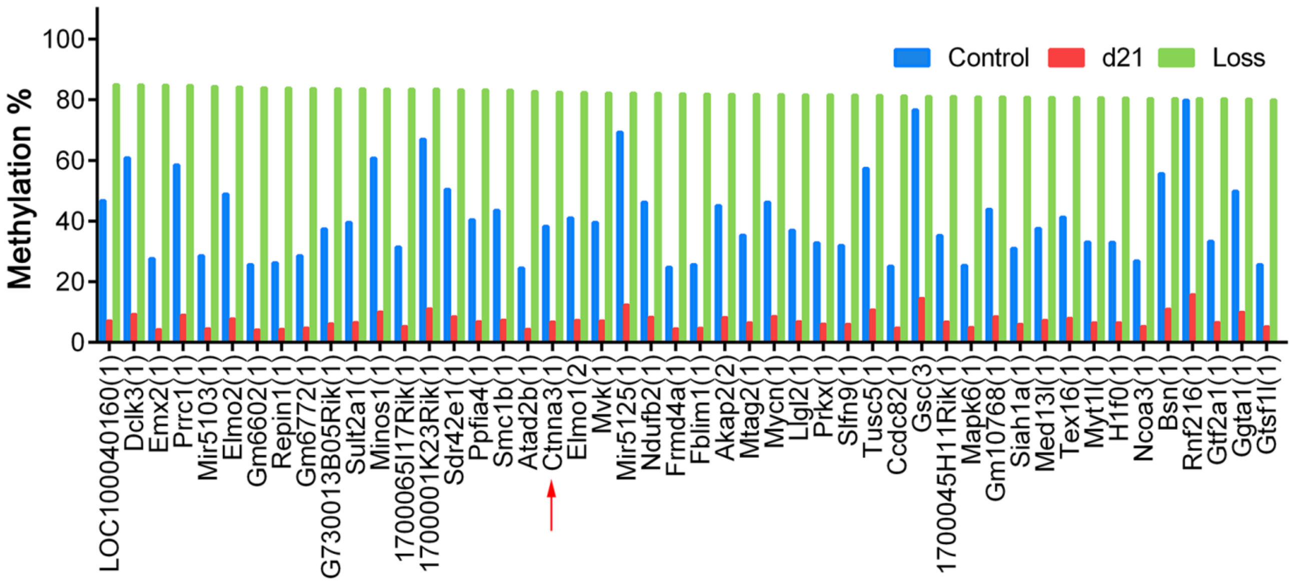

Figure S15, related to Figure 6. Classification by methylation reduction of annotated tiles in control vs d21 *Dnmt1$^{tet/tet}$* ES cells. Candidates showing a methylation loss ranging from 85% to 80% at d21. Bars represent the average of methylation averages of tiles associated to unique gene IDs. The number of annotated tiles for each gene ID is indicated in brackets. Known mouse imprinted genes are depicted by red arrows.

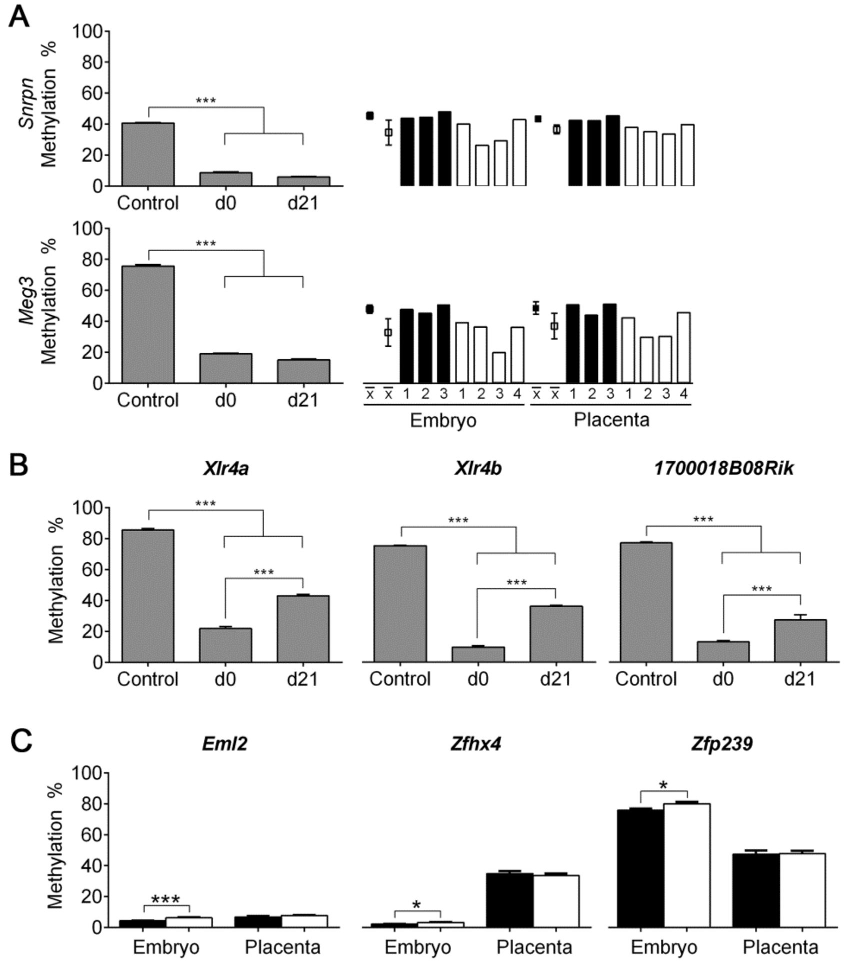

Figure S16, related to Figure 7. Imprinted genes and heritable non-gDMD candidates revealing methylation loss in *Dnmt1$^{tet/tet}$* ES cells at d21, as well as in embryos and placentae lacking methylation maintenance by *Dnmt1o*. DNA methylation quantification following the temporary suppression of *Dnmt1* (grey bars: control, d0 and d21) in *Dnmt1$^{tet/tet}$* ES cells and in 9.5dpc embryos and placentae from control (black bars) and *Dnmt1$^{\Delta 1o/\Delta 1o}$* mothers (white bars). A) Established imprinted gDMDs. For embryos and placentae, averages (x) and values for single samples are represented. B-C) Candidates regions. Quantified by pyrosequencing. *** p<0.005, ** p <0.01, * p<0.05.

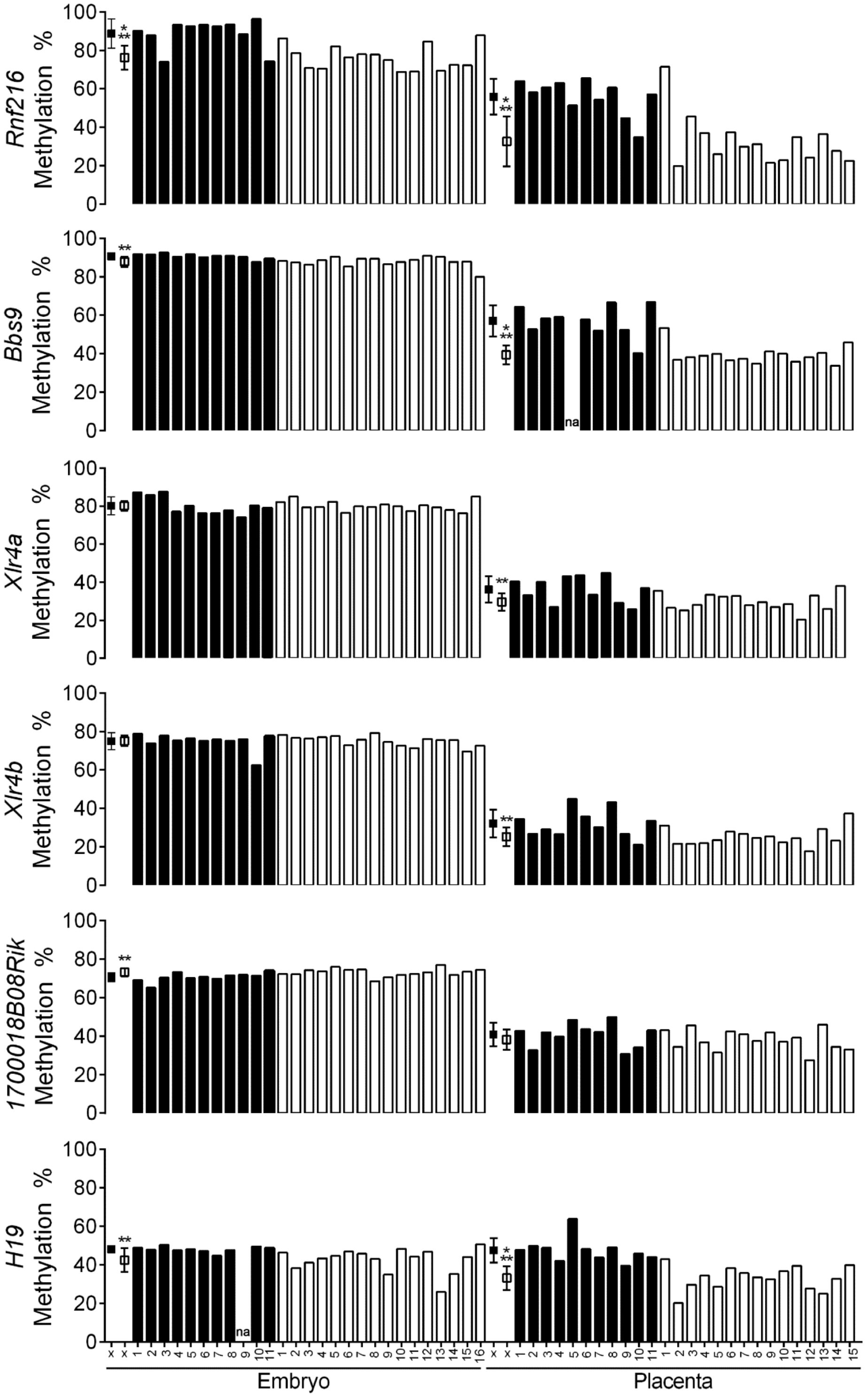

Figure S17, related to Figure 7. Heritable candidates revealing methylation loss in embryos and placentae lacking methylation maintenance by *Dnmt1o*. DNA methylation measurement of candidate regions in each single 9.5dpc embryos and placentae from control (black bars) and *Dnmt1*$^{\Delta 1o/\Delta 1o}$ mothers (white bars). Averages for each sample group are also shown. Quantified by pyrosequencing. *** p<0.005, ** p <0.01, * p<0.05.

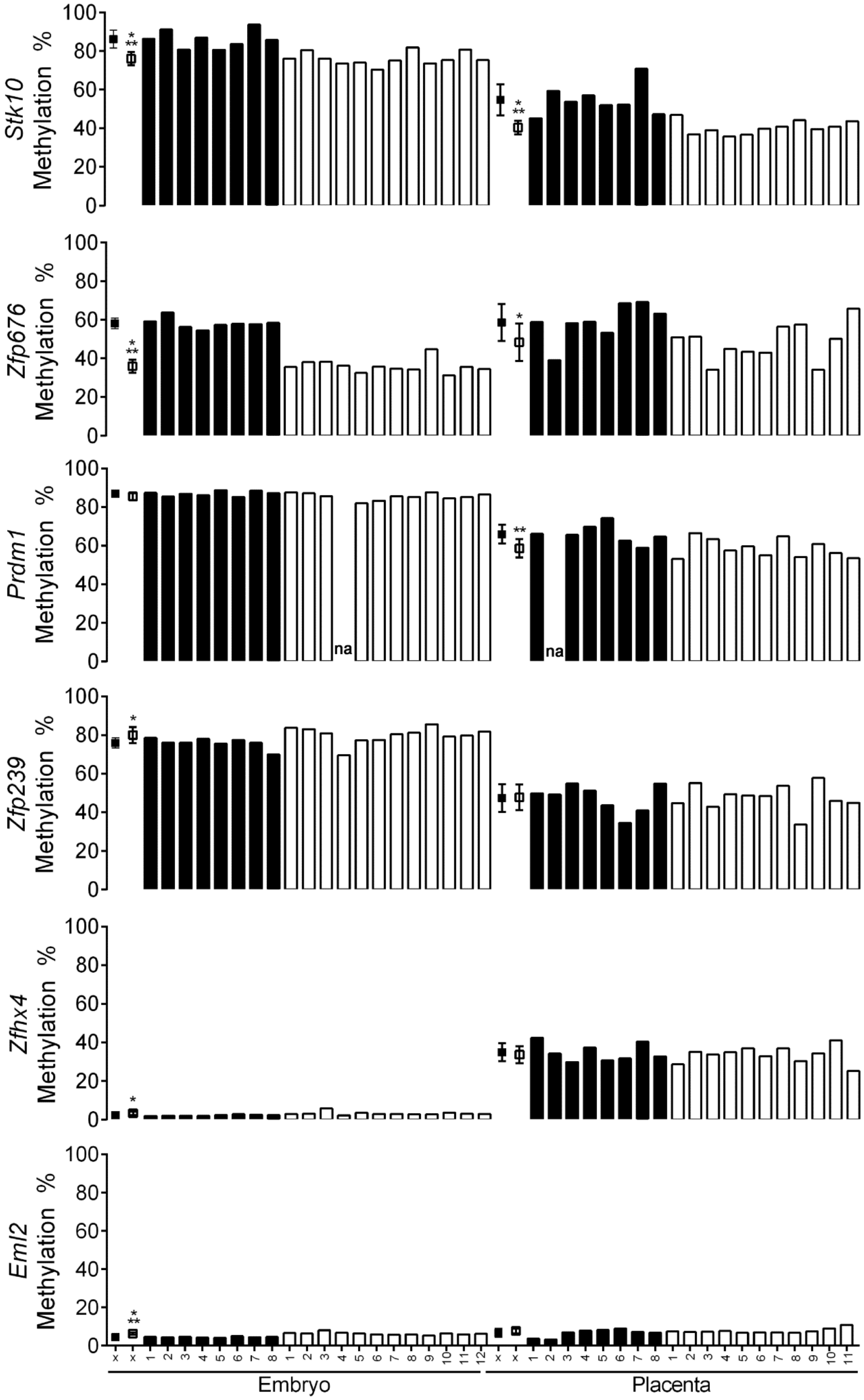

Figure S18, related to Figure 7. Heritable candidates revealing methylation loss in embryos and placentae lacking methylation maintenance by *Dnmt1o*. DNA methylation measurement of candidate regions in each single 9.5dpc embryos and placentae from control (black bars) and *Dnmt1*$^{\Delta 1o/\Delta 1o}$ mothers (white bars). Averages for each sample group are also shown. Quantified by pyrosequencing. *** p<0.005, ** p <0.01, * p<0.05.

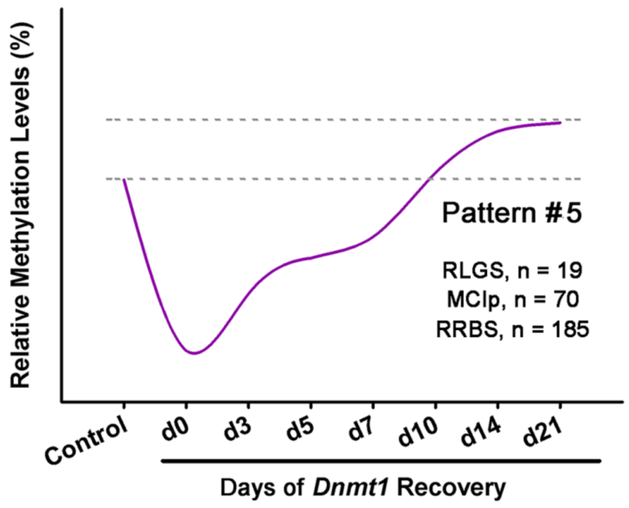

Figure S19, related to Supporting Text. Hypermethylation pattern at d21 following the interruption and reactivation of methylation maintenance by *Dnmt1* in *Dnmt1*$^{tet/tet}$ ES cells. RLGS analysis revealed that the transient suppression of *Dnmt1* generated a hypermethylation pattern of single copy loci *in Dnmt1*$^{tet/tet}$ ES cells (n=19)**.** This methylation increase at d21 was also observed by the MCIp (n=70 probes) and RRBS (n=185 tiles) techniques. "n" equals the number of observation with increase of methylation at d21 compared to original levels in controls.

.

A Top 20 MCIp candidates showing incrase DNA Methylation following the transient supression of *Dnmt1* in *Dnmt1 tet/tet* ES cells

Gene/Region	CGI Context	Location +/- 5000 bp	Array Intensity
Ethe1/Zfp575	Promoter/Inside	chr7:24580990-24591034	2.08
2810429I04Rik	Inside	chr13:3473530-3483574	1.82
Miip/Fv1	Divergent Promoter	chr4:147863702-147873761	1.58
AK180540	mRNA	chr10:78014129-78024173	1.58
Zfp869	Promoter	chr8:69711942-69721989	1.44
Arhgef25	Promoter	chr10:127184959-127195003	1.40
Zfp872	Promoter	chr9:22183260-22193304	1.29
Ogfod2	Inside	chr5:124107527-124117571	1.19
RN45s	Inside	chr17:39841644-39851688	1.15
Ppfia3	Promoter	chr7:45334663-45344707	1.09
Atxnil	Promoter	chr8:109732857-109742901	1.04
AK172441	mRNA	chr5:33438369-33448413	0.89
AK131965	mRNA	chr13:65255097-65265142	0.83
Ttll4	Inside	chr1:74657042-74667086	0.83
Dnmt1	Promoter	chr9:20947883-20957927	0.77
Epb4.1	Inside	chr4:132043961-132054005	0.75
Ethe1	Downstream	chr7:24606458-24616502	0.74
Gphn	Inside	chr12:78221866-78231910	0.73
Tcp11	Promoter	chr17:28075628-28085672	0.72

B

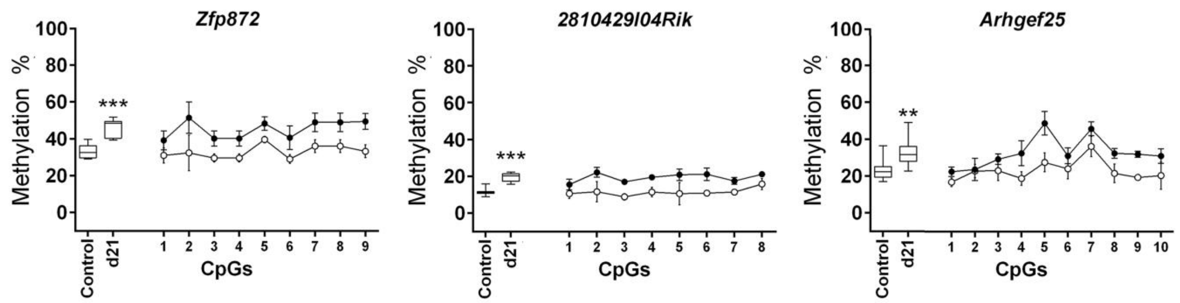

Figure S20, related to Supporting Text. Regions identified by a combination of MCIp enrichment and array hybridization revealing gain of DNA methylation following the inactivation and reactivation of *Dnmt1* in the *Dnmt1*$^{tet/tet}$ ES cells system. A) Characteristics of top array probes revealing gain of methylation at d21 vs control *Dnmt1*$^{tet/tet}$ ES cells. **B)** Hypermethylated regions analyzed for quantitative CpG methylation levels. Methylation levels are shown as averages ± SD, Ctrl (n=5) vs d21 (n=6). Open symbols are controls and filled symbols are d21. Levels of methylation at single CpGs were quantified by Sequenom MassArray. Control vs d21 *Dnmt1*$^{tet/tet}$ ES cells: *** p<0.005, ** p<0.01.

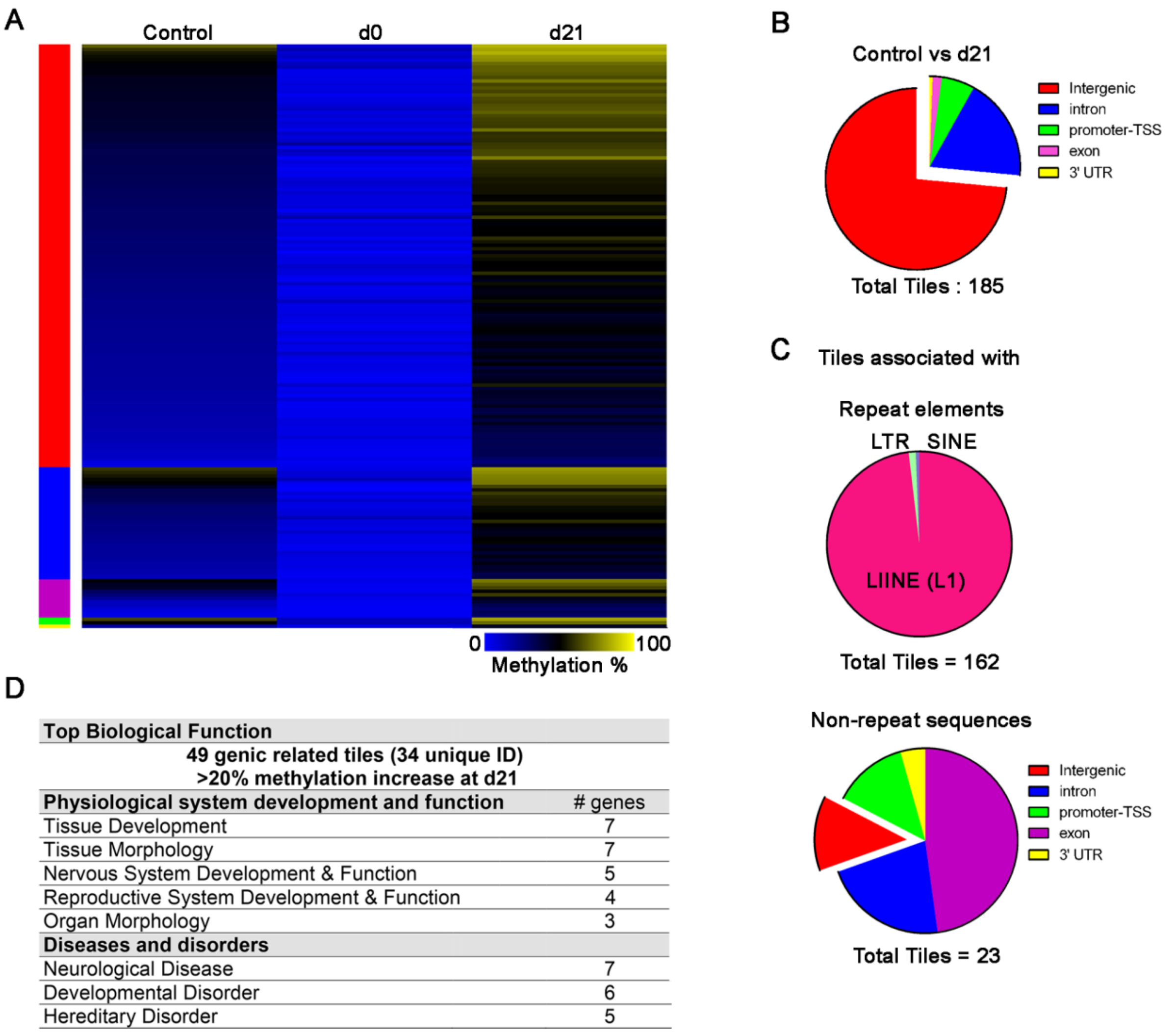

Top Biological Function	
49 genic related tiles (34 unique ID) >20% methylation increase at d21	
**Physiological system development and function**	# genes
Tissue Development	7
Tissue Morphology	7
Nervous System Development & Function	5
Reproductive System Development & Function	4
Organ Morphology	3
**Diseases and disorders**	
Neurological Disease	7
Developmental Disorder	6
Hereditary Disorder	5

Figure S21, related to Supporting Text. Genomic regions that had increased methylation levels subsequent to the interruption and reactivation of *Dnmt1 in Dnmt1*$^{tet/tet}$ ES cells. A) Heatmap representation of methylation levels for the 185 tiles that lost methylation at d0 and did not recover original levels by day 21. Methylation levels were clustered by genomic regions and by intensity displayed in control *Dnmt1*$^{tet/tet}$ ES cells. **B)** Distribution of annotated sequences features**. C)** Categorization of the 34 genic associated tiles by gene type. **D)** Summary of biological functions associated to the genic related tiles that showed increased methylation levels at day 21. Unique gene identification was use for biological function association.

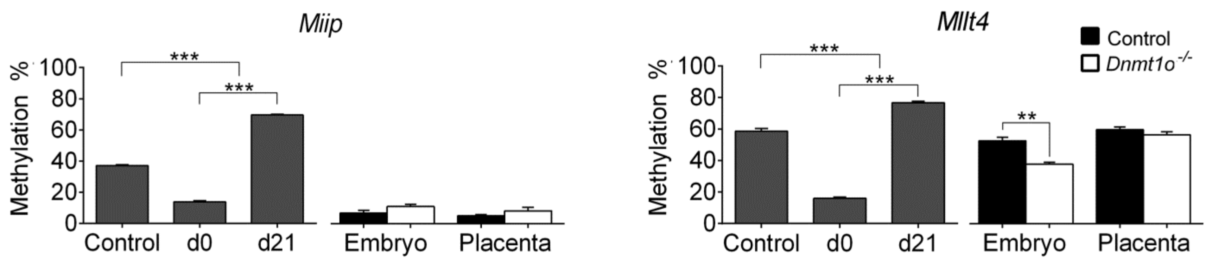

Figure S22, related to Supporting Text. Hypermethylation candidates revealing no significant methylation increase in embryos and placentae lacking *Dnmt1o*. Validation of RRBS candidate regions that gain DNA methylation following the temporary suppression of *Dnmt1* (grey bars). The methylation of these regions was quantified in 9.5dpc embryos and placenta from control and *Dnmt1*$^{\Delta 1o/\Delta 1o}$ mothers. Quantified by pyrosequencing. Control vs d21 *Dnmt1*$^{tet/tet}$ ES cells: *** $p<0.005$, ** $p<0.01$.

Table S5. List of primer sets and sequences used in PCR based experiments.

Target Regions	Forward (5'-3')	Reverse (5'-3')
**Sequenom MassArray**		
LINE-1	aggaagagagGGGTTTTTAATGAAGGAGTTAGAGAA	cagtaatacgactcactatagggagaaggctCCCTAACATTCCCCTATACTAAAACA
IAP	aggaagagagGTGAGTAATTGTTATTATAAGATGG	cagtaatacgactcactatagggagaaggctTTACTCACCCATCACCCCA
SINE	aggaagagagGTATTTGGGAGGTAGAGGTAGG	cagtaatacgactcactatagggagaaggctTCCTAAAACTCACTTTATAAACCAAA
Minor Satellite	aggaagagagTGAGTTATAATGAGAAATATGGAAAATGA	cagtaatacgactcactatagggagaaggctTCATTAATATACACTATTCTACAAATCCC
Gamma Satellite	aggaagagagTGAGAAATGTATATTGAAAGATTTGGA	cagtaatacgactcactatagggagaaggctAAACATTTCTAAATTTTCCACCTTTT
H19	aggaagagagGGTTTTATGAAGTTTATGATTATGGGA	cagtaatacgactcactatagggagaaggctAAAAATTCTACAAAAAAACCATACCC
Plag1	aggaagagagGTTAGTTAAGGGGATGGTTGTATGG	cagtaatacgactcactatagggagaaggctCAAATTTTCAAACTAAATTAAAACTACC
Gnas	aggaagagagTTGTTGAGAGGGTAGAGTTTAAGGA	cagtaatacgactcactatagggagaaggctACCAACAACTTTAAAAACTTTTCTATTC
Mest	aggaagagagGGTATTAATATATGGGAAGGTTTTTTT	cagtaatacgactcactatagggagaaggctCAACAAAAACAACAAACAACAACTC
Kcnq1	aggaagagagTGTAAGTTTGGGTTATAAAGATGGG	cagtaatacgactcactatagggagaaggctAAATTCTCTCAATTTTTCTTCAACA
Mico1	aggaagagagATAAGTGTTGTGGTTTGTTATGGGT	cagtaatacgactcactatagggagaaggctCCCTCACTCCAAAAATTAAAAAAAA
Intergenic Chr12	aggaagagagGGTTGGATAATGGGAGGTTTTTAG	cagtaatacgactcactatagggagaaggctAACCCCAAACCAAAAACAAATAC
Zfp787	aggaagagagTGGTTTTGATAGTTTTGGGTGTAAT	cagtaatacgactcactatagggagaaggctCCAATCTACACCTCCTCCTAAACC
Zfp872	aggaagagagGTTGTTATTTTGAGATTTGAGTGAGG	cagtaatacgactcactatagggagaaggctCCCCAAAAAACCTACCTAAAATAAA
2810429I04Rik	aggaagagagTGGGTTATTTTGTTTTTTGTTTTTG	cagtaatacgactcactatagggagaaggctACCTCCAAAATCAACACCATTC
Arhgef25	aggaagagagGTGGAGTGTAATTTTTTTAAAGTTTTTT	cagtaatacgactcactatagggagaaggctAAAAAAAACAACCCTACCCCTTTTA

Target Regions	Forward (5'3')	Reverse (5'-3')	Sequencing
**Pyrosequencing**			
Xlr4a	TGAAAGTTAGTGATAGGTAAAGGGTTA	Biot-CTAAACTCTCACTTCAACCTAAAAC	TATATTTTATTTATTATTTTTTATA
Bbs9	GTATTATTAGTGTGTATTGAAATAAGGGT	Biot-AAAAACAAACCACCCAAAACTAT	GTGTATTGAAATAAGGGTTT
Rnf216	AGTTAATTGGGATGGGGGAAT	Biot-CTACACAACCAACAAAAAACAAAACTAT	GAAAAGATGTTATTGATGA
Xlr4b	AGATTGTGGTGAGGTAGTG	Biot-AATCCAAATCATCACTTCACAAATTAAC	GTGTTAGAGGAAGGTG
1700018B08Rik	AGTTATGTTTGAAGGTTTGTTTTTGATAT	Biot-ACACATACCCAACACAATACA	ATTTTTAAGGTTGGAAAGAG
Prdm1	GTTTTTGGTTTATGGGATGGAGAGA	Biot-CATAACCAAAATAAAAACCCAAACCCTC	TTTAAGAGTTTTAGTTTTTATAGTA
Zfp676	GTTTTAGTTGGAGTTTTAATTTTATGT	Biot-CACACCTAACCCTAACAACT	GTTTTTATAGTGGTTTAAAGTTTTT
Stk10	AGTATGGGGGAGTTGGGT	Biot-CTACCCACCAAAAAAACTTTCT	GGGGGAGTTGGGTAA
Zfhx4	TGGTTATAAAGTAGTTAGTGAGGTATATG	Biot-AAAAACTCCAAACAAAAACCATAAT	GGAGATTTTTAGTTGGTTTTTT
Zfp239	TGTGGTAGGGTTTTTATTGGTTAAT	Biot-CCCCACACTTATAAAACCTCT	AAAGTTTTATATTTATAAGAGAGT
H19	GGGGGGTAGGATATATGTATTTTT	Biot-ACCTCATAAAACCCATAACTATAAAATCAT	GTGTGTAAAGATTAGGG
Myo5b	AGAGTTATGTAAGGTTGATAGAGT,	Biot-AAAACCAAAATTACACTTTCTTATTCC	GGTTGATAGAGTGGTGA
Gpi1	AGGGATATATAGTTATAGTAGTTAGTA,	Biot-ACACTATCACCCTCCTATATCAT	AGTTTTAGTATGGTTTATTTATTG
Wdfy3	GGGTTTTTTGAGAATAGTTGTGAGG	Biot-CAACCTACAACAATCAATCAACTTAACTTA	TGTGAGGTTGAGAAG
Stox1	ATAGATATTAAGTGGAGGAGGGTAT	Biot-CCCAACTATATTAACAACAAAACAACATCA	TGAATTGAAGTAAGAGTTTG
Itga9	TAGGATGTTGTGATAAATTATGGTATT	Biot-TAACTTAAAACCCCAAAACACAATACTCC	GGGTGGTGAGAGAG
Miip	GGTTAATGGGATTTTATTGTTGATTGG	Biot-TCTCTAACCCCACCTATCCACTA	TTTGGTTGGTTAGTAGTAT
Mllt4	AGTTTAGGGTTGATAGTATTAGTGG	Biot-AACATCAAAACACTTTCCCTAACTT	GGTTTGTTTAAAGAAGAAAGT
